# Deciphering the role of UBA-like domains in intraflagellar distribution and functions of myosin XXI in *Leishmania*

**DOI:** 10.1371/journal.pone.0232116

**Published:** 2020-04-28

**Authors:** Rani Bajaj, Bindu Ambaru, Chhitar M. Gupta

**Affiliations:** 1 Institute of Bioinformatics & Applied Biotechnology, Bengaluru, Karnataka, India; 2 Manipal Academy of Higher Education, Manipal, Karnataka, India; University of Heidelberg Medical School, GERMANY

## Abstract

Myosin XXI (Myo21) is a novel class of myosin present in all kinetoplastid parasites, such as *Trypanosoma* and *Leishmania*. This protein in *Leishmania* promastigotes is predominantly localized to the proximal region of the flagellum, and is involved in the flagellum assembly, cell motility and intracellular vesicle transport. As Myo21 contains two ubiquitin associated (UBA)-like domains (UBLD) in its amino acid sequence, we considered it of interest to analyze the role of these domains in the intracellular distribution and functions of this protein in *Leishmania* cells. In this context, we created green fluorescent protein (GFP)-conjugates of Myo21 constructs lacking one of the two UBLDs at a time or both the UBLDs as well as GFP-conjugates of only the two UBLDs and Myo21 tail lacking the two UBLDs and separately expressed them in the *Leishmania* cells. Our results show that unlike Myo21-GFP, Myo21-GFP constructs lacking either one or both the UBLDs failed to concentrate and co-distribute with actin in the proximal region of the flagellum. Nevertheless, the GFP conjugate of only the two UBLDs was found to predominantly localize to the flagellum base. Additionally, the cells that expressed only one or both the UBLDs-deleted Myo21-GFP constructs possessed shorter flagellum and displayed slower motility, compared to Myo21-GFP expressing cells. Further, the intracellular vesicle transport and cell growth were severely impaired in the cells that expressed both the UBLDs deleted Myo21-GFP construct, but in contrast, virtually no effect was observed on the intracellular vesicle transport and growth in the cells that expressed single UBLD deleted mutant proteins. Moreover, the observed slower growth of both the UBLDs-deleted Myo21-GFP expressing cells was primarily due to delayed G2/M phase caused by aberrant nuclear and daughter cell segregation during their cell division process. These results taken together clearly reveal that the presence of UBLDs in Myo21 are essentially required for its predominant localization to the flagellum base, and perhaps also in its involvement in the flagellum assembly and cell division. Possible role of UBLDs in involvement of Myo21 during *Leishmania* flagellum assembly and cell cycle is discussed.

## Introduction

Myosins are a diverse group of actin-based motor proteins, which are involved in several cellular activities, such as cell motility, intracellular trafficking, cytokinesis, muscle contraction, etc. [1]. These proteins are composed of a heavy chain, which consists of N-terminal head domain (motor domain) that contain actin and ATP binding sites and also possesses ATPase activity, a light chain (calmodulin)-binding neck domain, and a C-terminal tail domain that imparts functional specificity to different classes of myosins [2]. Based on the high degree of sequence conservation in the head domain, myosins have been anticipated to power their movements along F-actin tracks, whereas the variable tail domain facilitates the cargo binding and transportation. Most eukaryotic organisms require myosins for a variety of their cellular functions, but a few taxonomic groups, such as red algae and diplomonads appear to live without them [3]. Based on variations in their amino acid sequence and domain composition, myosins have been classified into more than >30 classes in different organisms [4] some of which fall into the category of novel class of myosins. For example, kinetoplastid parasites such as *Trypanosoma brucei*, *Trypanosoma cruzi* and *Leishmania* all contain one class I myosin and one myosin belonging to a novel class of myosins, class XXI. In addition to these two myosins, *T*. *cruzi* contains additional six more myosins, five of which fall into class XXI, whereas TcMyo8 still remains unclassified [5,6]. Interestingly, the tail region of kinetoplastid myosins contains protein domains that were not known earlier to be associated with myosins, e.g., FYVE, WW, or UBA (Ubiquitin associated)-like domains [5].

*Leishmania* are a group of eukaryotic parasites that cause several human diseases, including cutaneous and visceral leishmaniasis, and have digenetic life cycle, requiring an insect vector (sand fly) and animal host for their multiplication and propagation [7]. Within the sand fly vector, *Leishmania* exist extracellularly in form of promastigotes, which have elongated cell body and long motile flagellum, whereas in animals, these parasites reside and multiply within the macrophages adopting the amastigote morphology with a rounded cell body and barely visible flagellum. The genome of these organisms encodes for only two homologues of myosins, one belonging to class IB and the second belongs to class XXI [8]. Earlier studies have shown that *L*. *donovani* promastigotes express only class XXI myosin (Myo21), which predominantly localizes to the proximal region of the flagellum [9], and regulates the flagellum assembly, cell motility and intracellular trafficking [9,10]. It has further been reported that the prominent localization of Myo21 to the proximal region of the flagellum is not dependent on the myosin head region but it is almost exclusively determined by the myosin tail region [9]. As *Leishmania* Myo21, unlike other myosins, contains two putative UBA-like domains in its tail region, we considered it of interest to analyze whether these domains have any role in the intracellular distribution and functions of Myo21 in *Leishmania* promastigotes. Our results indicate that-(I) both the UBA-like domains are essentially required for prominent localization of Myo21 to the proximal region of the flagellum and flagellum assembly, and (II) only one of the two UBA-like domains may perhaps be sufficient to maintain the normal activity of Myo21 during intracellular vesicle transport and cell division.

## Materials and methods

Mouse monoclonal antibody against α-Tubulin (B-5-1-2; Cat. No.23948) was procured from Santacruz Biotechnology company, whereas mouse monoclonal antibodies against β-tubulin (Cat. No. T7816) and GFP (GF28R; Cat. No.MA5-15256) were purchased from Sigma and Invitrogen, respectively.

### *Leishmania* culture and growth analysis

*Leishmania donovani* promastigotes were routinely cultured in high glucose Dulbecco’s modified Eagle’s medium (DMEM; Gibco, Life Technologies) supplemented with 10% of heat inactivated fetal bovine serum (FBS; MP Biomedicals) and 40 mg L^-1^ gentamycin at 25°C. Cultures were allowed to reach stationary phase (6–7 days post inoculation), prior to inoculation into fresh medium. Myo21-GFP and other GFP chimera expressing cells were maintained in the presence of 100 μg mL^-1^ of G418 sulfate. For growth analysis, cells were inoculated at 10^5^ cells mL^-1^ density in the medium without antibiotic, and the cell number was counted every 24 h for up to 11 days with a hemocytometer.

### Myo21 gene expression and protein purification

Recombinant Myo21 was expressed and purified as described earlier [9]. Identity of the purified recombinant Myo21 was determined by western blotting, using antibodies against the histidine tag (His_6_).

### Generation of polyclonal rabbit antiserum against recombinant Myo21 and chicken antiserum against recombinant *Leishmania* actin

To raise polyclonal antiserum against Myo21, rabbits were injected with the purified protein (Outsourced to Geniron Biolabs Pvt. Ltd. Bangalore, India, paid service). The monospecific polyclonal antiserum against Myo21 from the immunized rabbit serum was prepared, as reported earlier [11]. Specificity of the antibodies was confirmed by western blotting of *Leishmania* cell lysate, wherein it specifically recognized only a single band of expected molecular weight of ~115 kDa.

*Leishmania* actin (LdAct) clone was obtained from Amogh Sahasrabuddhe (CDRI, Lucknow, India). Recombinant LdAct was expressed in *E*. *coli* and the protein from the inclusion bodies was purified, as reported earlier [12]. Antiserum against pure recombinant LdAct was raised in chickens (Paid Service, Geniron Biolabs Pvt. Ltd., India), and monospecific polyclonal anti-LdAct antisera was prepared by affinity chromatography, using the published procedure [12].

### Generation and expression of full length and truncated Myo21-GFP fusion constructs

Full length open reading frame of Myo21 (amino acid: 1–1050) was PCR amplified with primer pair F1/R1 (S1 Table) from the genomic DNA, and cloned in frame to the C–terminus GFP gene in pXG-GFP+ vector at BamH I site (Myo21-GFP). Truncated forms of Myo21, lacking both the UBA- like domains (amino acid: 1–953) and only the UBA2 domain (amino acids: 1–1011), were PCR amplified using primer pairs F1/ R2 and F1/R4 (S1 Table), respectively, and then ligated in pXG-GFP+ vector at BamHI site (Myo21ΔUBAs-GFP; Myo21ΔUBA2-GFP). To clone the Myo21 lacking only the UBA1 domain, two steps PCR was done, first two fragments were amplified using primer pairs F1/R3 (amino acids: 1–953) and F2/R1 (amino acids: 991–1050) (S1 Table). Both the fragments were gel eluted and used as a template for second step of overlapping PCR with primer pairs F1/R1 and finally cloned as described above. Cloning of Myo21 UBA like domains (both 1 and 2; GFP-Myo21UBAs) and Myo21 tail lacking both the UBA like domains (GFP-Myo21TΔUBAs) was done at Bam H I site in pXG-GFP2+ vector after their amplification using primer pairs F3/R5 (amino acids: 954–1050) and F4/R6 (amino acids: 751–953), respectively (S1 Table). The authenticity of each clone was confirmed by DNA sequencing. The clone was transfected in mid-log phase *Leishmania* promastigotes by electroporation and selection was done under increasing concentration of G418 sulfate (20–100 μg mL^-1^). This was followed by clonal selection for each construct in order to obtain homogeneous culture, but without much success, as they became heterogeneous after 2–3 generations.

### Western blotting

Mid-log phase *Leishmania* promastigotes (5–8 X 10^6^ cells) were used for the analysis. Lysates were prepared by washing the cells two times with phosphate buffer saline (PBS; pH 7.4) and boiling the re-suspended cells in SDS (sodium dodecyl sulfate)- polyacrylamide gel sample buffer in the presence of protease inhibitor (1mM PMSF, 1mM benzamidine hydrochloride hydrate and protease inhibitor cocktail (Roche)). Equal amounts of lysates were loaded and resolved on 10% SDS–polyacrylamide gels by electrophoresis. Gels were electro blotted on nitrocellulose membrane, followed by incubation of the membrane in blocking buffer (5% skimmed milk in TBS (Tris buffer saline; pH 7.4)). The membrane was first probed with primary antibodies (anti-GFP, 1:2500; anti-Myo21, 1:2500; anti-LdAct, 1:5000) diluted in blocking buffer. After removing the unbound antibodies by washing, the membrane was probed with HRP-conjugated secondary antibodies (HRP-conjugated anti-mouse IgG, anti-rabbit IgG or anti-chicken IgY; Invitrogen; 1:5000). Blots were developed by chemiluminescence ECL substrate (Amersham, GE healthcare) and imaged in a BioRad Molecular Imager® ChemiDoc^™^ XRS+ imaging system. Quantification of band intensity was done by Gel Quant.net software.

### Immunofluorescence microscopy

Cells were washed twice with PBS (pH 7.4) and allowed to adhere on poly–L–lysine coated coverslips for 5–10 min. Adhered cells were fixed in paraformaldehyde (2%, w/v) solution for 30 min, followed by washing with PBS–glycine (0.5%, w/v) solution. For visualization of GFP–tagged protein expression, coverslips were mounted in ProLong Diamond Anti-fade Mountant with 4, 6-diamidino-2-phenylindole, DAPI (Invitrogen; P36966) and images were captured on Nikon laser scanning confocal microscope c2 using a 100 x 1.4 NA (oil) lens. For co-labeling with antibodies, fixed cells were permeabilized by their treatment with PBS containing 0.5% (v/v) Triton X-100, and blocking was done with 3% bovine serum albumin (BSA) solution in PBS (w/v). The immunolabeling of cells was carried out, using anti-α/β tubulin (1:1000) or anti-LdAct as primary antibodies and anti-mouse Alexa Fluor 568 or anti-chicken Alexa Fluor 568 as secondary antibodies. Coverslips were mounted in ProLong Diamond Anti-fade Mountant with DAPI and imaging was done on Nikon laser scanning confocal microscope c2 using a 100 x 1.4 NA (oil) lens. Negative control slide was used to blank background signals and adjust the gain/offset and laser powers before the image data collection. Wherever required images were adjusted for brightness/contrast and were cropped and arranged for presentation in Adobe Photoshop.

For nuclear and kinetoplast configurations analyses, the cells from the mid–log phase were stained with DAPI and categorized into 1N1K, 2N2K, 2N1K, depending on the number of nuclei and kinetoplasts per cell and the percentage of each category was quantified in three independent experiments (n≥600) in each case.

### Intracellular vesicle transport

Endocytic vesicles were traced microscopically by using the fluorophore FM^™^4-64FX (Invitrogen) as described earlier [13]. Briefly, *Leishmania* cells (5–10 X 10^6^ cells mL^-1^) were incubated in culture medium with 10% FCS and 2 μg mL^-1^ FM^™^4-64FX for 10 min at 25°C in the dark, harvested and re-suspended in fresh medium. Small aliquots were withdrawn at various time intervals, washed twice with cold PBS before adhering onto poly-L-lysine coated coverslips. Adhered cells were fixed with 2% paraformaldehyde, washed and mounted in ProLong Diamond Anti-fade Mountant with DAPI and imaging was done on Nikon® ECLIPSE Ni-E microscope using a 100X (oil) lens.

### Motility assessment by time-lapse microscopy and video microscopy

Motility measurements were performed on a Nikon® ECLIPSE Ni-E microscope after collecting time-lapse movie for 30 seconds with a 40X objective. Paths of individual cells from the time-lapse movie were traced using MTrack2 tracking tool from Fiji (ImageJ). To analyze the motility rate, cells with complete path tracks were considered. Motility rate was determined by dividing the total path travelled by the time taken and plotted in the graph. For cell swimming analysis video at 100 frames per second were captured at 40 X magnification.

### Cell cycle analysis

For cell cycle analysis, the mid-log phase promastigotes (≤10^7^ cells mL^-1^) were synchronized with hydroxyurea (HU) as described earlier [14]. Cells were collected by centrifugation at 1400 X g for 10 min at 4°C, resuspended in fresh DMEM containing 200 μg mL^-1^ HU (Sigma), and the mixture was incubated overnight (12 h). After incubation, the cells were collected by centrifugation, washed two times with PBS and then re-suspended in fresh DMEM media without HU. Approximately 10^7^ cells were collected at every 2 h time interval for up to 12 h. The cell suspension was mixed with 150 μl of fixative solution (1% Triton X-100, 40mM citric acid, 20 mM sodium phosphate, 200 mM sucrose) and incubated for 5 min at room temperature. To the fixed cells, 350 μL of diluent buffer (125 mM MgCl_2_ in PBS) was added and the samples were stored at 4°C until further use. To measure DNA, the cells were first incubated with 50 μg RNase (5 mg mL^-1^ in 0.2M sodium phosphate buffer, pH 7.0) at 37°C for 2 h and then stained with 50 μg PI (5 mg mL^-1^ in 1.12% sodium citrate) at 25°C for 1 h followed by overnight equilibration at 4°C. The data was acquired in Gallios flow cytometer (Beckman coulter), and the proportions of G1, S and G2/M populations were determined using ModFit software. Around 20,000 events were collected for each sample.

### Quantification and statistical analysis

The data were statistically analyzed by ANOVA test provided in the Microsoft excel software. A p-value of >0.05 was considered significant.

## Results

**Fig 1:**
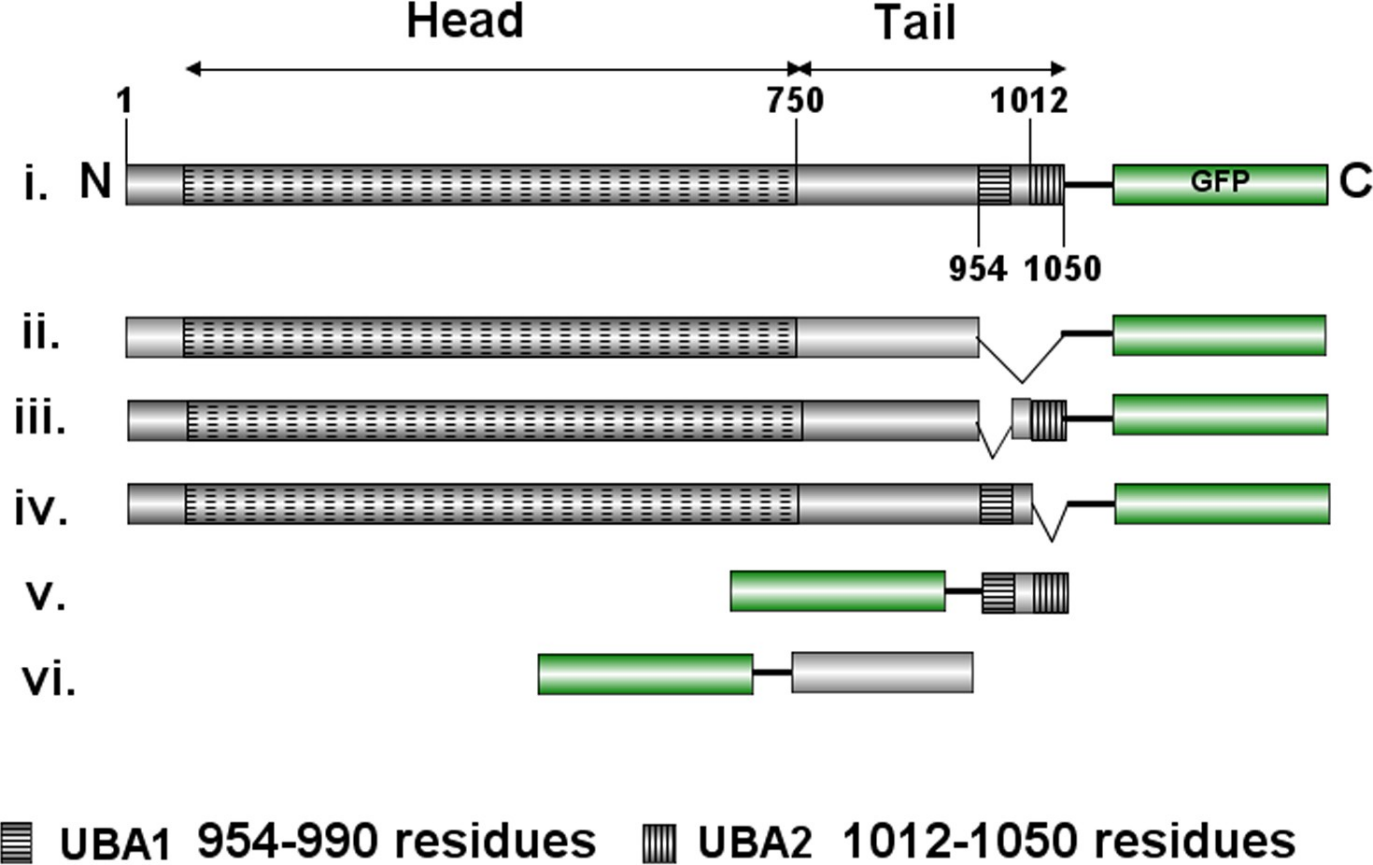
Design of constructs. Schematic diagram of the Myo21 protein sequence with domains indicated and various GFP fusion chimeras used in this study. The distal tail region contains two UBA-like domains, named as UBA1 and UBA2. The domains-deleted molecules used in the study have been named according to the region deleted, such as (i) Myo21-GFP, (ii) Myo21ΔUBAs-GFP, (iii) Myo21ΔUBA1-GFP, (iv) Myo21ΔUBA2-GFP, (v) GFP-Myo21UBAs, and (vi) GFP-Myo21TΔUBAs.

Myo21 was expressed in bacteria, purified, and then monospecific anti-Myo21 antibodies prepared, as reported by us earlier [9]. These antibodies specifically recognized a single band of molecular mass of about 115 kDa in whole cells lysate of *L*. *donovani* promastigotes (S1A, S2A and S2B Figs). In *Leishmania* cells, Myo21 besides being distributed throughout the cell body, it also concentrated predominantly at the proximal region of the flagellum, which is determined solely by its tail region [9]. Bioinformatic analysis (Conserved Domain Database; Pfam) of Myo21 amino acid sequence identified the presence of two putative UBA-like domains at its C—terminus spanning the tail region. UBA-like domains, in general, have been reported to be about 35–45 amino acid residues long, and are present in proteins that are involved in cell cycle regulation, DNA repair and ubiquitin/proteasome pathways [15]. In an effort to characterize the role of these domain/s in intracellular localization and functions of Myo21, Myo21-GFP fusion proteins were constructed to contain full length Myo21 (amino acids 1–1050, Myo21-GFP), the truncated Myo21 lacking both the UBA-like domains (amino acids 1–953, Myo21ΔUBAs-GFP), only one UBA-like domain at a time (amino acids 1-953/991-1050, Myo21ΔUBA1-GFP; amino acids 1–1011, Myo21ΔUBA2-GFP), only the two Myo21 UBA-like domains (amino acids 954–1050, GFP-Myo21UBAs) and Myo21 tail lacking both the UBA-like domains (amino acids 751–953, GFP-Myo21TΔUBAs) (Fig 1).

**Fig 2:**
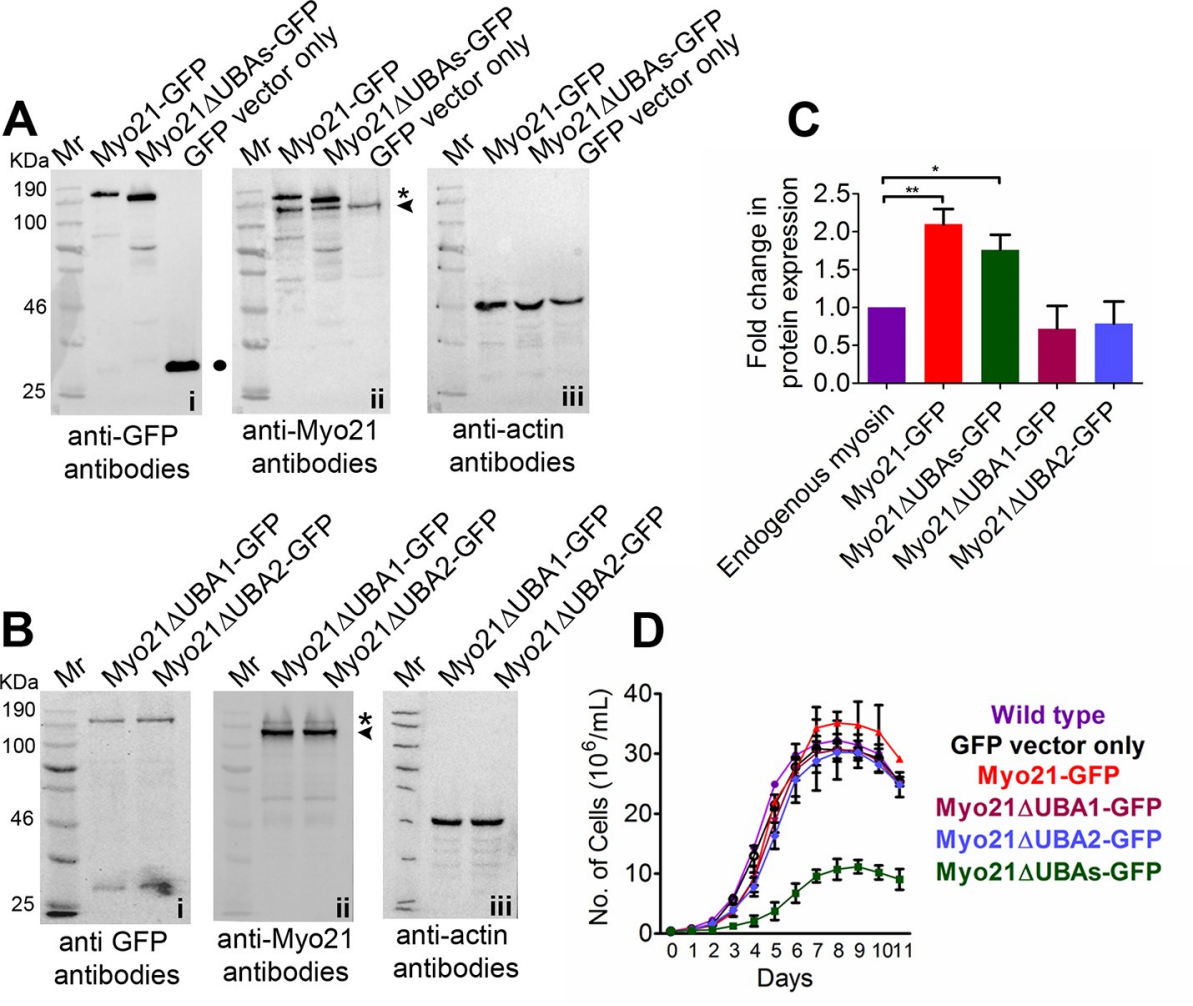
Protein expression and growth analyses. (A and B) Western blot analysis of *Leishmania* cell lysates expressing GFP fusion proteins (10^7^ cells equivalent). Blots probed with anti–Myo21 antibodies were stripped and re-probed with anti-GFP and anti-actin antibodies (Note—Additional bands visible on the blot are probably due to partial degradation of GFP-fused proteins, despite lysing the cells in the presence of protease inhibitors). Mr, molecular weight markers. (C) The GFP-tagged protein band (asterisk) signal intensity was quantified, normalized to endogenous Myo21 band (arrowhead) in the respective lane (GelQuant.net software) and fold change in expression was calculated. Data shown are means of three independent experiments ± S.D. **, p≤0.01; *, p≤0.04. S. D., standard deviation. (D) Growth analysis of wild type and mutant cells shows that the growth rate of Myo21ΔUBAs-GFP expressing promastigotes is severely compromised and the cells grew at a significantly lower density, as compared to wild type or Myo21-GFP expressing cells. The results are expressed as the means ±-S.D. of three independent experiments.

Each of these constructs was transfected separately in *Leishmania* promastigotes and the expression of fusion proteins was confirmed by western blotting, using anti-GFP and anti-Myo21 antibodies (Fig 2A & 2B; S1B, S1C, S1D and S2C Figs). Densitometry analysis revealed approximately two-fold expression of the Myo21-GFP and Myo21ΔUBAs-GFP, and about 1:0.7-fold expression of Myo21ΔUBA1-GFP and Myo21ΔUBA2-GFP proteins relative to the endogenous Myo21 (Fig 2C). Despite increasing the antibiotic G418 sulfate, concentration from 100 to 250 μg mL^-1^ in the culture medium, there was no further increase in expression levels of single UBA deletion constructs.

### Expression of Myo21ΔUBAs-GFP in *Leishmania* promastigotes severely impaired their growth in culture

The Myo21ΔUBAs-GFP expressing cells grew considerably slower (Mean generation time: 16 ± 1.7 h) than the Myo21-GFP expressing or wild type cells (Mean generation time: Myo21-GFP expressing cells—8.7 ± 0.7 h; wild type cells—9.3 ±1 h), never reaching the same cell density as the control cells (Fig 2D). Notably, we frequently observed rounded and some oversized cells in the late stationary phase cultures of Myo21ΔUBAs-GFP expressing cells. Further, GFP-Myo21UBAs expressing cells grew at higher cell density, whereas GFP-Myo21TΔUBAs expressing cells grew at slightly lower densities, compared to the control cells (S2D Fig). However, the expression of other truncated Myo21 proteins in the promastigotes had virtually no effect on their growth (Fig 2D), suggesting that only one UBA- like domain in Myo21 may perhaps be sufficient to maintain its activity in modulating the cell growth in culture, and that Myo21 could be involved in regulation of *Leishmania* cell cycle.

### The C-terminus region harboring UBA-like domains is essentially required for flagellum base localization of Myo21

**Fig 3:**
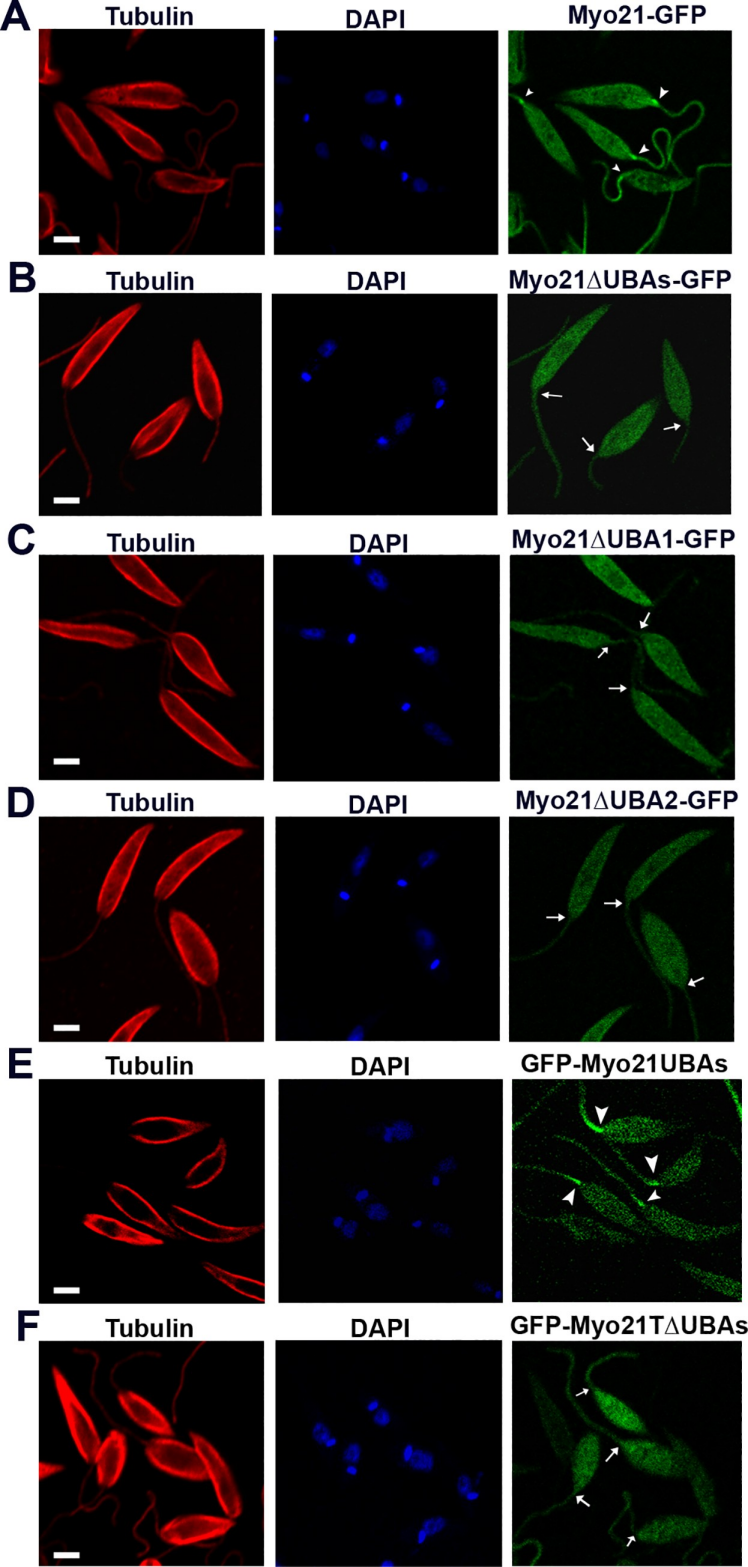
UBA-like domains of Myo21 are crucial for flagellar base localization of Myo21. Confocal microscopy images of *Leishmania* promastigotes expressing (A) Myo21-GFP, (B) Myo21ΔUBAs-GFP, (C) Myo21ΔUBA1-GFP, (D) Myo21ΔUBA2-GFP, (E) GFP-Myo21UBAs, and (F) GFP-Myo21TΔUBAs. Cells were labeled for α-tubulin (red) and mounted in DAPI (blue) to visualize the DNA (nucleus and kinetoplast). In contrast to Myo21-GFP (n = 85), Myo21ΔUBAs-GFP (n = 159), Myo21ΔUBA1-GFP (n = 123), Myo21ΔUBA2-GFP (n = 140) and GFP-Myo21TΔUBAs (n = 93) distributed throughout the cell body but lacked predominant localization to the proximal region of the flagellum. However, unlike these proteins, the construct containing GFP-conjugate of only the two UBA-like domains of Myo21 appeared to prominently localize to the flagellum base in most cells (>75%; n = 87) that expressed this protein. Arrowheads indicate localization of GFP-tagged protein to the proximal region of the flagellum in ‘A’ and ‘E’ and arrows indicate lack of such localization in ‘B, C, D & F’. Scale bar—2 μm.

The intracellular distribution of the expressed proteins was monitored by immunofluorescence/fluorescence microscopy. The results revealed that similar to Myo21-GFP, Myo21ΔUBAs-GFP was distributed throughout the cell body except that it failed to predominantly localize to the proximal region of the flagellum (Fig 3A & 3B; S3A & S3B Fig). Like Myo21ΔUBAs-GFP protein, Myo21ΔUBA1-GFP and Myo21ΔUBA2-GFP proteins also failed to concentrate at the base of the flagellum (Fig 3C & 3D; S3C & S3D Fig), suggesting that both the UBA-like domains might have been required for prominent localization of Myo21 to the proximal region of the flagellum. To examine the validity of this suggestion, we separately transfected the *Leishmania* cells with GFP-Myo21UBAs and GFP-Myo21TΔUBAs constructs (S1D & S2C Figs) and then analyzed their intracellular distribution by confocal microscopy. Results given in Fig 3E and 3F clearly reveal that in most GFP-Myo21UBAs expressing cells (>75%; n = 87), the construct appeared to prominently localize to the proximal region of the flagellum, whereas no such intraflagellar distribution could be seen in GFP-Myo21TΔUBAs expressing cells (n = 93). These results clearly demonstrate that the presence of both the UBA-like domains in Myo21 is essential for its prominent localization to the flagellum base.

As Myo21 has been reported to partially co-localize with actin [10], we analyzed the association of these mutant proteins with actin, using immunofluorescence microscopy. As expected, in Myo21-GFP expressing cells, Myo21-GFP co-distributed with actin in the cell body and also at the proximal region of the flagellum (S4A Fig). However, no such intraflagellar co-distribution of actin could be seen in cells expressing other mutant proteins (S4B, S4C & S4D Fig).

### Both the UBA-like domains in Myo21 are required for its role in regulation of the cell morphology, flagellum length and motility

**Fig 4:**
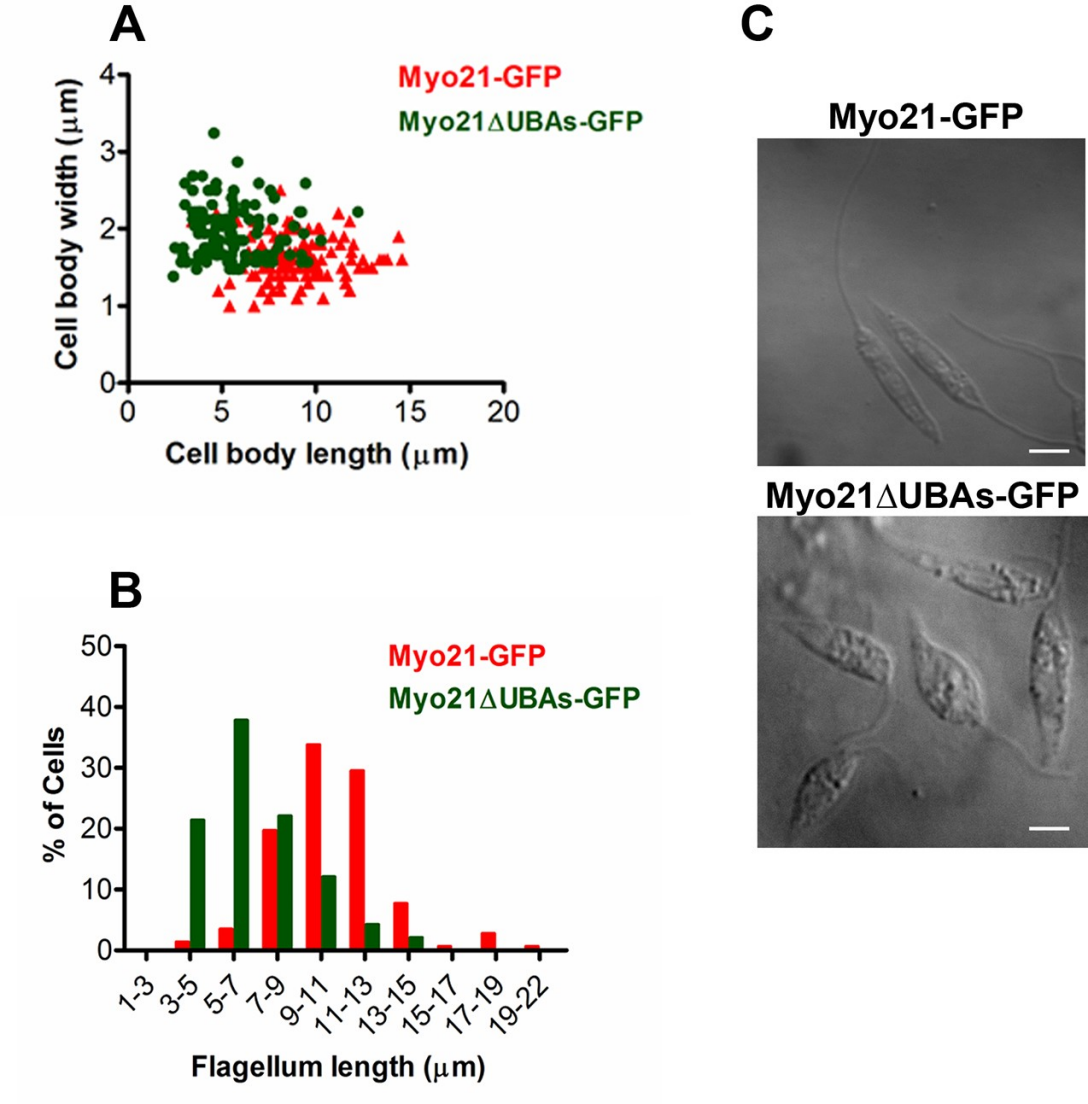
Expression of Myo21ΔUBAs-GFP alters the morphology of *Leishmania* cells. (A) Correlation of cell body length and width of Myo21-GFPand Myo21ΔUBAs-GFP cells. (B) Histogram of flagellum lengths of Myo21-GFP and Myo21ΔUBAs-GFP cells. More than 120 1N1K cells were measured from about 30 different fields in at least three independent experiments for each cell type. ***p≤0.0006 for body length, ***p≤0.0003 for body width and ***p≤0.0008 for flagellum length. The data were statistically analyzed by ANOVA test and a p-value of >0.05 was considered significant. (C) Representative images of Myo21ΔUBAs-GFP expressing cells, showing altered morphology and shortened flagellum length, compared to Myo21-GFP expressing cells. Scale bar—2 μm.

Our earlier studies have shown that Myo21 regulates the cell morphology, motility, flagellum length and intracellular vesicle transport in *Leishmania* promastigotes [10]. To examine whether UBA-like domains have any role in these Myo21 functions, we analyzed the cell body length, width, flagellum length and motility of *Leishmania* cells expressing various truncated Myo21-GFP constructs. Whereas expression of Myo21-GFP in wild type cells virtually had no effect on their cell morphology and flagellum length (wild type cells–mean body length: 9.5 ± 1.2 μm, mean body width: 1.44 ± 0.36 μm, mean flagellum length: 10.7 ± 3 μm; Myo21-GFP expressing cells–mean body length: 9.0 ± 2.0 μm, mean body width: 1.6 ± 0.27 μm, mean flagellum length: 10.5 ± 2.5 μm; S5A & S5B Fig), a large number of stumpy cells possessing shorter flagella were seen in cultures of cells that expressed Myo21ΔUBAs-GFP (Myo21ΔUBAs-GFP expressing cells- mean body length: 6 ± 1.5 μm, mean body width: 2.2 ± 0.4 μm, mean flagellum length: 5.0 ± 2.2 μm; Fig 4A, 4B & 4C).

**Fig 5:**
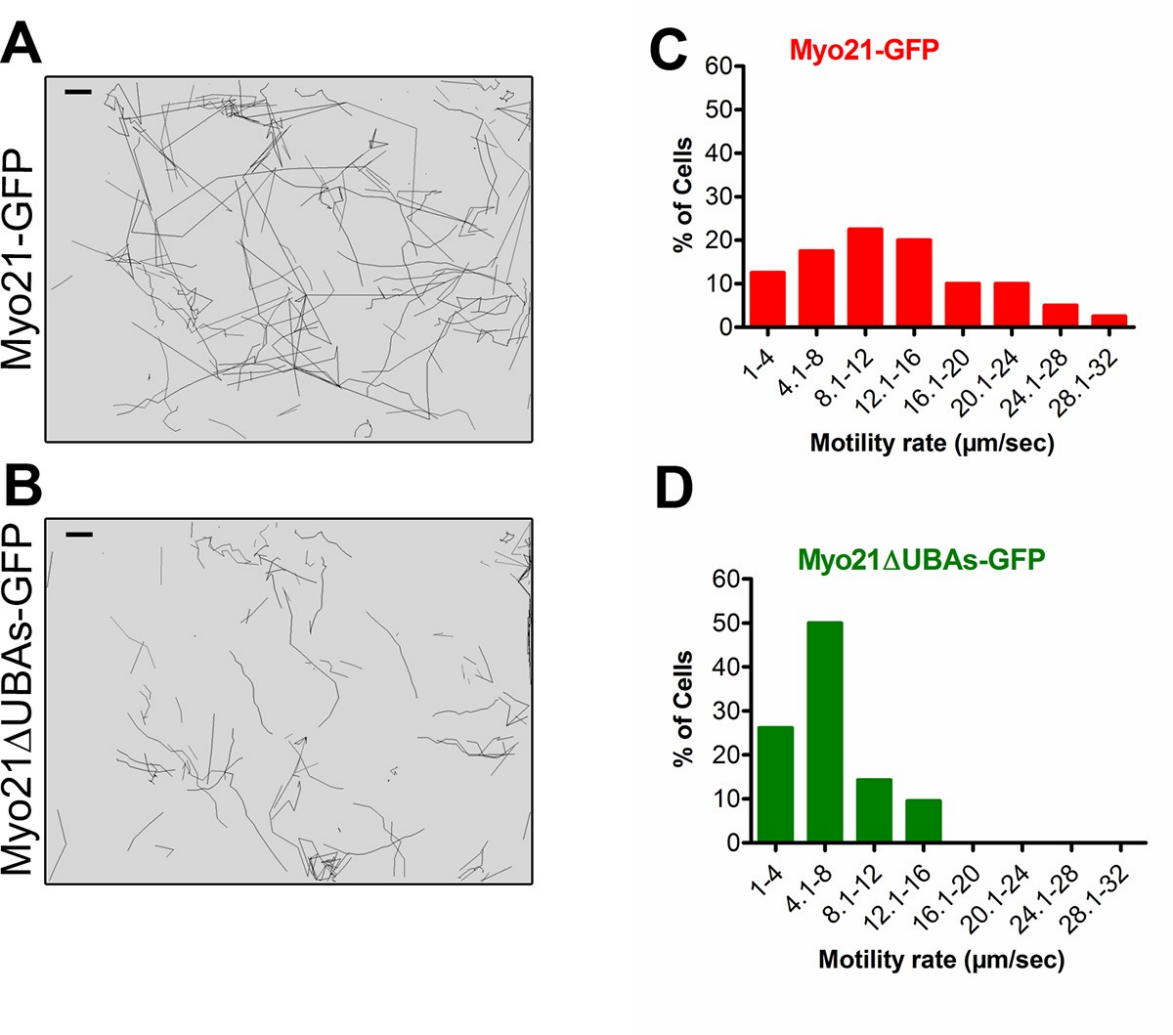
Expression of Myo21ΔUBAs-GFP alters the motility of *Leishmania* cells. Swimming tracks from time-lapse movie of parasites expressing (A) Myo21-GFP and (B) Myo21ΔUBAs-GFP tracked using MTrack2 tracking tool in Fiji (ImageJ). Scale bar—100 μm. Motility rate was determined by dividing the total path travelled by the time taken and plotted in the graph for Myo21-GFP (C; n = 42) and Myo21ΔUBAs-GFP (D; n = 40) expressing cells from at least three independent experiments; **p≤0.003. The data were statistically analyzed by ANOVA test and a p-value of <0.05 was considered significant.

As *Leishmania* cell motility is directly influenced by the flagellum length [10, 16, 17], we measured motility of cells expressing Myo21-GFP and Myo21ΔUBAs-GFP by video microscopy (Fig 5A, 5B, 5C & 5D; S1 and S2 Movies, respectively) in at least three independent experiments. Results revealed that Myo21ΔUBAs-GFP expressing cells mostly swam at slower rate and their average motility rate was significantly less than of Myo21-GFP expressing cells (Myo21-GFP expressing cells–Mean motility: 12.7 ± 4 μm/sec, n = 40; Myo21ΔUBAs-GFP expressing cells–Mean motility: 6.58 ± 1.9 μm/sec, n = 42; **p≤0.003).

The cells expressing individual UBA domain deleted constructs possessed slightly reduced body length and increased body width, compared to control cells (Myo21ΔUBA1-GFP expressing cells - body length: 7.8 ± 1.2 μm, body width: 1.6 ± 0.3 μm; Myo21ΔUBA2-GFP expressing cells-body length: 7.5 ± 1.2 μm, body width: 1.8 ± 0.3 μm; S5C Fig). Although the mean flagellum length of these cells was nearly comparable to the control cells (Myo21ΔUBA1-GFP expressing cells—9.62 ± 2.1 μm; Myo21ΔUBA2-GFP expressing cells—8.4 ± 1.6 μm); however, an appreciable number of these cells possessed a relatively shorter flagellum (S5D Fig). Further, video microscopy analysis revealed that average motility rate of these cells was slightly reduced, as compared to the control cells (Mean motility rate of Myo21ΔUBA1-GFP expressing cells: 10.8 ± 1.8 μm/sec, n = 33 and Myo21ΔUBA2-GFP expressing cells: 9.6 ± 0.5 μm/sec, n = 36; S6A, S6B, S6C, S6D & S6E Fig; S3 & S4 Movies). These results clearly indicate that UBA-like domain(s) in Myo21 are required for its involvement in regulation of *Leishmania* promastigote morphology, flagellum length and motility.

### Only one UBA-like domain in Myo21 may be sufficient to maintain its normal activity in intracellular vesicle trafficking

**Fig 6:**
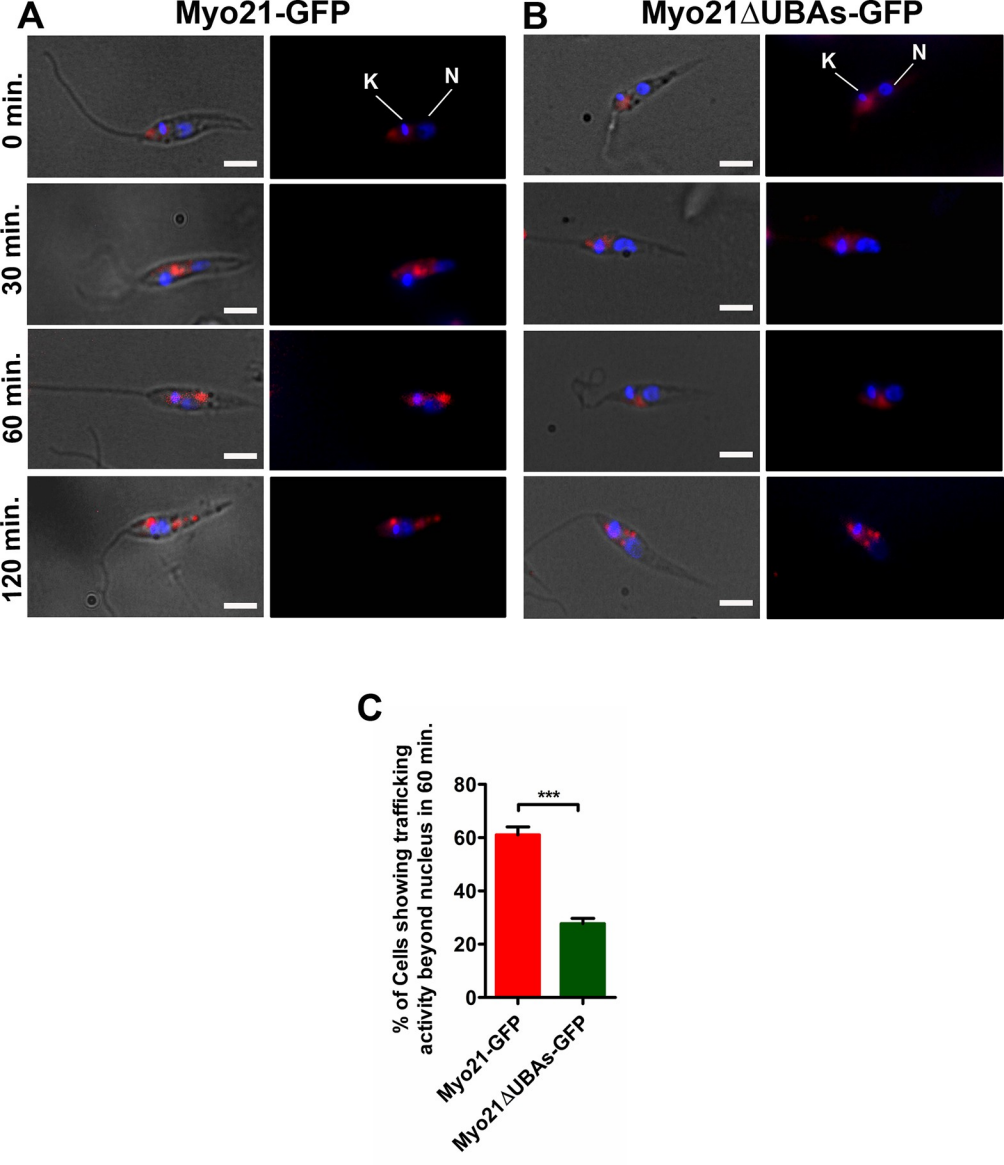
Myo21ΔUBAs-GFP expressing cells have reduced rate of vesicular trafficking. Uptake and intracellular transport of FM4-64FX fluorescent dye in (A) Myo21-GFP expressing cells, and (B) Myo21ΔUBAs-GFP expressing cells. Cells were incubated with FM4-64FX (marker for endosome) for 10 min and then washed and suspended in fresh medium. Measured aliquots of cells were taken out at 0 min, 30 min, 60 min and 120 min. Adhered and fixed cells were stained with DAPI (blue) to visualize the nucleus (N) and the kinetoplast (K); FM4-64 dye is in red. Scale bar—2 μm. (C) Quantitative analyses of Myo21-GFP and Myo21ΔUBAs-GFP expressing cells that trafficked FM4-64 dye beyond the nucleus in 60 min (n = 67 and 53 for Myo21-GFP and Myo21ΔUBAs-GFP expressing cells, respectively, from three independent experiments; ***p≤0.0008). The data were statistically analyzed by ANOVA test and a p-value of <0.05 was considered significant.

To examine whether expression of GFP-conjugates of UBA-like domain deleted Myo21 constructs in *Leishmania* cells has any effect on the intracellular trafficking activity of Myo21, we analyzed the uptake and intracellular transport of a fluorescent hydrophobic dye FM4-64 in the cells, separately expressing various GFP conjugates of Myo21 deletion constructs, using Myo21-GFP expressing cells as the control. In the control cells, the dye was efficiently endocytosed and trafficked down from the flagellar pocket region to the posterior end at 25°C in 60 min (Fig 6A), whereas in identical conditions, the dye in most cells expressing Myo21ΔUBAs-GFP construct did not cross the nuclear region (Fig 6B). In 60 min, only 28% Myo21ΔUBAs-GFP expressing cells trafficked the fluorophore beyond the nucleus (n = 53 from three independent experiments) as opposed to 68% Myo21-GFP expressing cells (n = 67 from three independent experiments; Fig 6C). However, expression of Myo21ΔUBA1-GFP or Myo21ΔUBA2-GFP construct in *Leishmania* promastigotes exhibited no effect on the vesicular trafficking activity of Myo21, as about 66% or 61% cells of respective cell type trafficked the fluorophore beyond the nucleus (n = 43 and 36 for Myo21ΔUBA1-GFP and Myo21ΔUBA2-GFP, respectively; from three independent experiments S7A, S7B & S7C Fig). From these results we infer that only one UBA-like domain may be sufficient to maintain the normal activity of Myo21 in intracellular vesicle trafficking.

### Impaired cell division in Myo21ΔUBAs-GFP expressing cells is due to delayed G2/M phase in cell cycle

**Fig 7:**
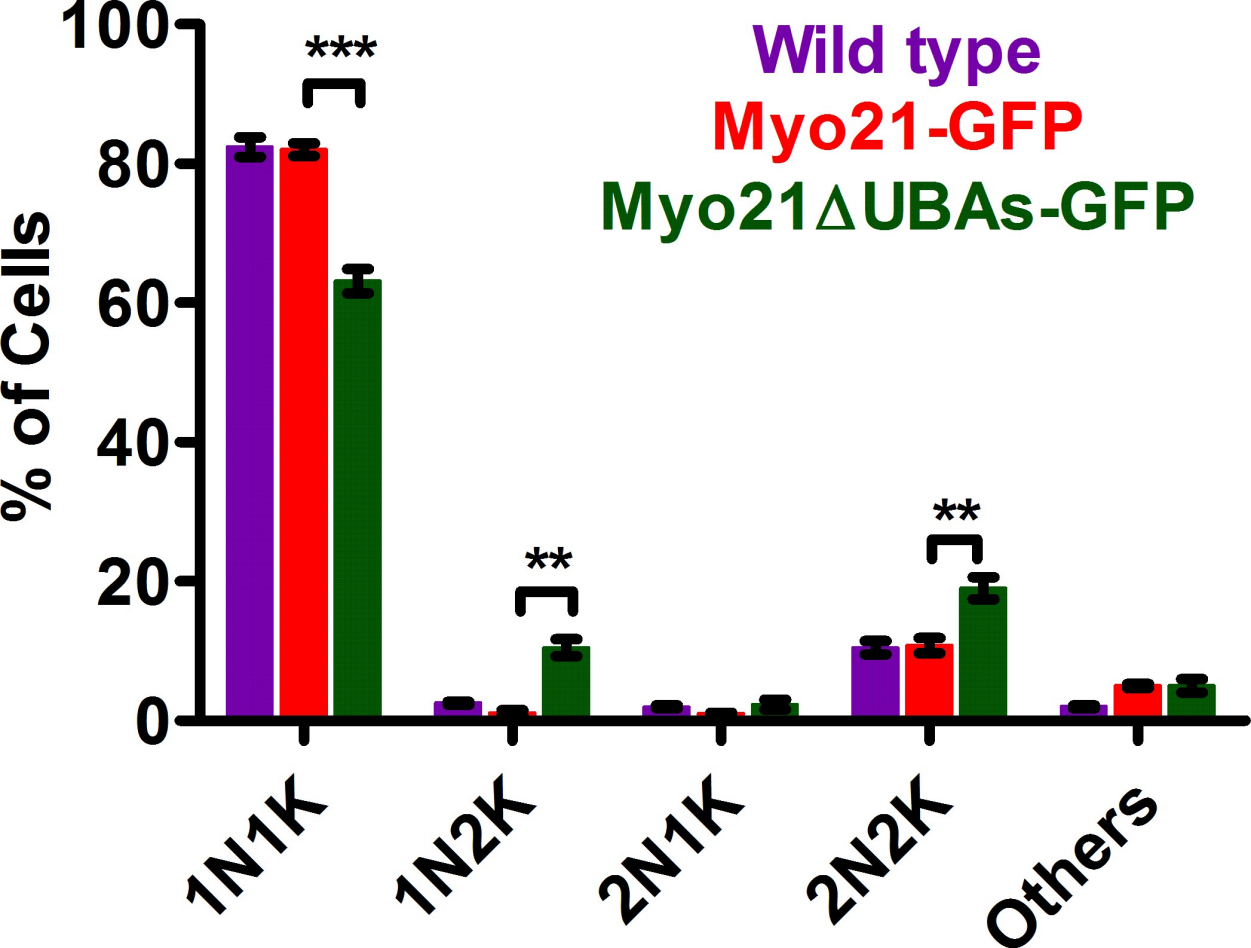
Cell configurations according to the number of nuclei and kinetoplasts in *Leishmania* cells, Myo21-GFP and Myo21ΔUBAs expressing cells. Quantification of DNA contents of DAPI stained mid-log phase wild type cells (purple bar), Myo21-GFP expressing cells (red bar), and Myo21ΔUBAs-GFP expressing cells (green bar) by microscopy. Cells were categorized depending on the number of nucleus and kinetoplast present (1N1K, 1N2K, 2N1K, 2N2K & others) and the results from an average of three independent experiments are plotted in the graph (n≥600). There is significant reduction in 1N1K counts (***p≤0.001) and increase in 1N2K (**p≤0.01) and 2N2K counts (**p≤0.01) in Myo21ΔUBAs-GFP expressing cell population, compared to control cells. The data were statistically analyzed by ANOVA test and a p-value of <0.05 was considered significant.

Next, the possible link between the increased generation time and perturbation of one or more stages during cell division cycle of Myo21ΔUBAs-GFP expressing cells was probed by assessing the position in cell division cycle of an individual cell in culture. In *L*. *major*, tracking the distribution of DNA containing organelles (Nucleus, N; Kinetoplast, K) allows assessment of the cell cycle position of individual cells within a population [18]. During the initiation of cell cycle, a cell contains only one N and one K (1N1K). Subsequently, the kinetoplast divides first (1N2K) followed by division of nucleus, resulting in 2N2K cells and finally cytokinesis produces two daughter 1N1K cells. Detailed examination revealed that the frequency of cells with 1N2K and 2N2K configuration increased significantly in Myo21ΔUBAs-GFP expressing cell cultures, as compared to the control cell cultures (Fig 7), indicating aberrations in nuclear and daughter cell segregation.

**Fig 8:**
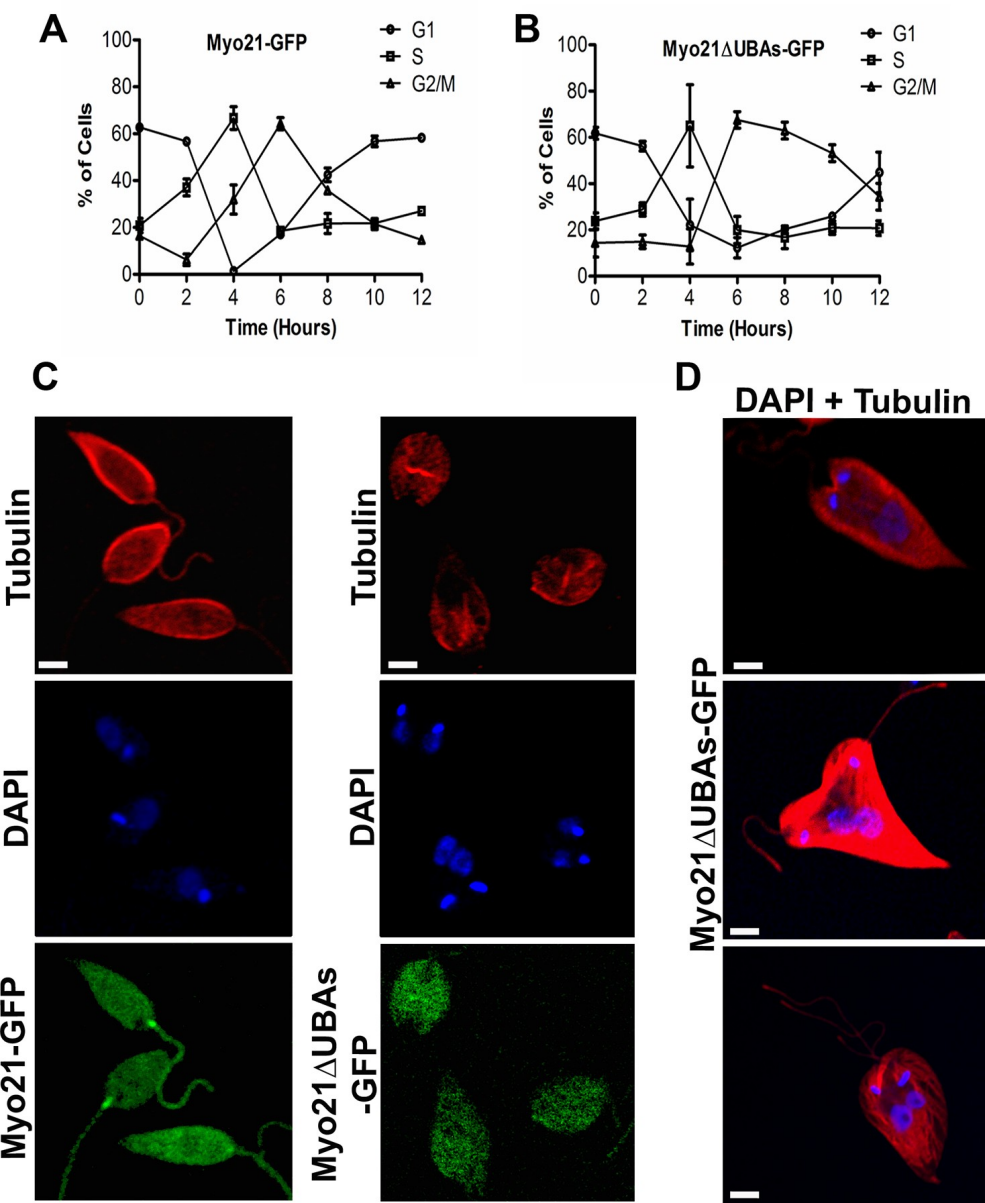
A prolonged G2/M phase slows down the cell cycle progression of Myo21ΔUBAs-GFP expressing cells. Graphical representation of cell cycle of (A) Myo21-GFP expressing cells, and (B) Myo21ΔUBAs-GFP expressing cells, after removal of hydroxyurea (HU) block. Mid-log phase cells were synchronized by the HU treatment. DNA content was measured after staining the cells aliquots, taken out at 2 h interval up to 12 h, with propidium iodide (PI) and analyzing the stained cells by flow cytometry. G1 phase—circle, S phase—square, and G2/M phase—triangle. The results shown are means ± S.D. of three independent experiments. (C) Representative fluorescence microscopy fields showing the presence of higher number of dividing Myo21ΔUBAs-GFP expressing cells. Cells were treated with HU overnight and samples were collected at 8 h after removal of HU block. The washed cells were labeled with α-tubulin antibodies (red) and DAPI (blue). Most Myo21ΔUBAs-GFP expressing cells were arrested at the 2N2K stage. (D) Representative images of gigantic sized Myo21ΔUBAs-GFP expressing cells, showing either bulky nucleus or connected nuclei (DAPI-blue). Myo21-GFP expressing cells, n = 84 and Myo21ΔUBAs-GFP expressing cells, n = 103 from three independent experiments. Scale bar—2 μm.

To validate the function of UBA -like domain in cell division, we probed the cell cycle progression after synchronizing the cells at G1/S transition by hydroxyurea (HU) treatment. After releasing the HU block, measured aliquots of the cells were collected at 2 h interval up to 12 h, and stained with propidium iodide (PI) to monitor the cell cycle progression from G1 through S and G2/M, and back into G1 by flow cytometry. About 80% of Myo21-GFP expressing cells and Myo21ΔUBAs-GFP expressing cells were arrested in the G1-S phase after HU treatment. The Myo21-GFP expressing cells entered the S phase at 4 h, G2/M phase at 6 h and completed it by 8 h to re-enter into the next G1 phase. Similarly, Myo21ΔUBAs-GFP expressing cells also entered the G2/M phase at 6 h, but unlike the Myo21-GFP expressing cells, they took significantly longer time (12 h) to navigate through the G2/M phase before entering G1 phase (Fig 8A & 8B; S8 Fig). The flow cytometry profiles of Myo21-GFP and other mutant protein (viz. Myo21ΔUBA1-GFP and Myo21ΔUBA2-GFP) expressing cells were similar to the wild type cells (S9 & S10 Figs). These results suggest that the UBA-like domains of Myo21 may be involved in steering out the cells through the G2/M phase of cell cycle.

### Delayed G2/M phase progression results from mitotic and cytokinetic arrest

To ascertain whether the observed G2/M arrest is due to delayed mitosis or post-mitotic, Myo21ΔUBAs-GFP expressing cells were labeled with DAPI, 8 h after removal of HU block and then analyzed for the nucleus and kinetoplast division (Fig 8C). Results revealed increased accumulation of 1N2K and 2N2K Cell population (1N2K = 16.5%, 2N2K = 46.4%; n = 103 from at least three independent experiments) in Myo21ΔUBAs-GFP expressing cells, as compared to Myo21-GFP expressing cells (1N2K = 7%, 2N2K = 23%; n = 84 from at least three independent experiments). A large proportion (about 76%) of Myo21ΔUBAs-GFP expressing cells having 1N2K configuration were gigantic in size, several fold larger than the normal size cells. These cells contained either the connected nuclei or a bulky nuclear aggregate, indicating mitotic inhibition (Fig 8D). Further, 11% of these gigantic cells displayed uninterrupted kinetoplast division and blocked cytokinesis. Besides, 45% of 2N2K mutant cell population did not possess cleavage furrow, suggesting that the delayed G2/M phase in Myo21ΔUBAs-GFP expressing cells is partly due to mitotic arrest and largely post-mitotic (cytokinesis).

## Discussion

Myo21 is an actin—based motor [19], which regulates the flagellum assembly, cell motility, and intracellular vesicular trafficking in *Leishmania* promastigotes [10]. As this protein contains two UBA-like domains towards the end of its tail region [9, 20], and usually the tail region determines functions of myosins within the cell [2], we considered it of interest to elucidate the role of these domains in Myo21 functions. For this, we expressed various truncated Myo21 constructs fused with GFP in *Leishmania* promastigotes, and then observed the dominant negative effects of their expression on Myo21 functions. Similar approach has been used earlier by several other workers to analyze the specific roles of myosins in cells. For example, myosin-1C from *Dictyostelium discoideum* is known to simultaneously interact with F-actin and microtubules in the region close to the spindle poles and the cell cortex, and also to regulate the spindle stability for accurate chromosome separation. However, expression of full-length myosin-1C with point mutation in its motor domain, which reduced its affinity for actin, exhibits dominant negative effects on the spindle morphology and results in enlarged nucleus and prolonged mitosis [21]. Further, expression of dominant negative tail construct has been reported to identify a group of six myosins (XIC, XIE, XIK, XI-I, MYA1, and MYA2) out of 17 myosins, that are more important for motility of the Golgi bodies and the mitochondria in Nicotiana benthamiana and Nicotiana tabacum [22–24]. Furthermore, GFP-conjugate of a truncated fragment of the non-muscle myosin II-A heavy chain (NMHC II-A) lacking amino acids 1–591, DN592, has been used to examine the cellular functions of this protein in HeLa cells [25]. Besides, a dominant negative approach using GFP-Myo5c construct has been followed to ascertain the functions of Myo5c in HeLa cells [26]. In addition, the dominant negative effects of the GFP-Myo5b tail chimera revealed that this myosin homologue in HeLa or MDCK cells is required for transit of plasma membrane recycling systems [27]. Finally, expression of brush border myosin I truncated in the motor domain impairs the distribution and functions of endocytic compartments in hepatoma cell line [28], and affects membrane traffic in polarized cells [29].

The Ubiquitin-Associated (UBA) domains are approximately 35–40 amino acids long and are known to be present in proteins associated with ubiquitylation and DNA nucleotide excision-repair [15]. These domains bind ubiquitin, multi-ubiquitin chains, ubiquitylated proteins and other effectors, predicting a role for these domains in protein-protein interactions and subcellular targeting [30]. It has been reported that UBA and UBA-like domain containing proteins associate with the substrates that are destined for degradation and also with subunits of proteasome, and thereby regulate the proper turnover of proteins in the cell [31]. Further, the C-terminal UBA domains have been shown to protect ubiquitin receptors by preventing initiation of protein degradation at the proteasome [32]. As Myo21 contains two UBA-like domains towards the end of the tail region, we envisaged that these domains could play an important role in Myo21 functions during the flagellum assembly and the cell cycle regulation.

The flagellum, in general, is a highly dynamic microtubule-based structure, which imparts motility and sensory functions associated with a wide range of biological processes [33–36]. Unlike other flagellated organisms, such as *Chlamydomonas*, where the flagellum is comprised of only one component, the axoneme [37], the flagellum of kinetoplastid parasites is composed of two components, the axoneme and the paraflagellar rod (PFR). The canonical 9 + 2 axoneme structure powers beating in most eukaryotic flagella [38], whereas the PFR imparts flagellar motility and waveform generation [39]. The dynamics of the flagellum involves a process of assembly and disassembly, which requires movements of protein cargoes from the flagellum base to its tip (anterograde) and also from the tip to the base (retrograde), which is termed as ‘intraflagellar transport’ (IFT) [40]. IFT is mainly powered by the microtubule-based motor proteins, such as kinesin II and dynein complexes for anterograde and retrograde transports, respectively [41]. However, besides the microtubules-based motor proteins, the actin-based motor Myo21 has also been implicated in the assembly and dynamics of the *Leishmania* flagellum, especially the PFR [10]. Further, it has earlier been suggested that the proteins that are released upon flagellum disassembly are presumably transported back to the cytoplasm, where they get rapidly degraded [42, 43]. Consistent to this suggestion, it has recently been shown that during the disassembly of the *Chlamydomonas* flagellum at least 20 proteins get polyubiquitylated prior to their transport to the cytoplasm for degradation [37]. Here, we propose that during *Leishmania* flagellum disassembly, Myo21 by virtue of the presence of two UBA-like domains at its C-terminus [32], may shuttle the released proteins after their presumed ubiquitylation, for degradation by the ubiquitin-proteasome pathway [44], without getting itself degraded. This suitably accounts for the observed similar effects of single and double UBA-like domain deleted Myo21-GFP constructs on the flagellum assembly and motility. Further, since Myo21 tail region, including UBA-like domains, is known to nonspecifically bind to anionic phospholipids [20], it is likely that this protein might have been serving as a lipid transporter during assembly and disassembly of the flagellum membrane.

The cell division cycle is driven by a large number of enzymes, which coordinate DNA replication and chromosome segregation. The activity of the enzymes that perform and coordinate these biological processes oscillates by regulated expression and/or posttranslational modifications. ubiquitylation is a versatile modification that has been used to fine-tune these cell cycle events, frequently through processes that do not involve proteasomal degradation [45]. Aurora kinases are the key enzymes involved in regulation of normal chromosome segregation during mitosis and cytokinesis in all eukaryotic cells. The ubiquitylation/deubiquitylation of these enzymes is known to finely control their activation and deactivation during their role in mitosis and cytokinesis [46]. Further, aurora kinase 1, AUK1, has been reported to play a critical role in mitosis and progression of cytokinesis in both procyclic and bloodstream forms of *T*. *brucei* [47–49] as well as in *Leishmania* cells [50]. As myosins are also known to be involved in these processes in eukaryotic cells [21, 51, 52], we speculate that during *Leishmania* cell division cycle, Myo21 through its UBA- like domain (s) perhaps associates with ubiquitylated form of aurora kinase to regulate the mitotic and cytokinetic processes in these cells. This is consistent with our current observation that these processes are adversely affected in cell division cycle of the Myo21ΔUBAs-GFP- expressing cells, however, no such problem is encountered during cell division in the Myo21ΔUBA1/ UBA2-GFP expressing cells.

Finally, this study shows that the two UBA-like domains present towards the end of the tail region in Myo21 mostly determine its prominent localization to the flagellum base. As this region in Myo21 has been reported to contain six potential nonspecific lipid-binding sites, out of which two sites fall within the aa 953 –aa 1050 region, which includes the region that contains both the UBA-like domains [20], we envisage that the binding of these domains with flagellar membrane lipids may perhaps be responsible for prominent localization of Myo21 at the proximal region of the flagellum.

## Supporting information

S1 Fig The original, uncropped and unadjusted images underlying all blots and gels. (TIFF)

S2 Fig Generation and specificity of antibodies against *Leishmania M*yo21 protein. (A) Commassie stained SDS-polyacrylamide gel (10%) showing over-expressed Myo21 protein in bacterial cell lysate after IPTG induction and purified recombinant Myo21 (rMyo21) protein (~115KDa, arrowhead). Lane 1: Uninduced bacterial cell lysate, Lane 2: Induced bacterial cell lysate, Lane 3: rMyo21; Mr: molecular weight markers. Purified Myo21 protein was used for generation of antibodies. (B). Monospecific polyclonal Myo21 antibodies purified from rabbit serum were validated by western blotting, which detects a specific band of expected molecular weight ~115 KDa in *Leishmania* cell lysate (arrowhead). (i) Commassie stained SDS-polyacrylamide gel (10%). (ii) Western blot of using purified Myo21 antibodies. Lane 1: *Leishmania* cell lysate, lane 2: rMyo21; Mr: molecular weight markers. (C) Expression of Myo21UBAs-GFPand Myo21TΔUBAs-GFP in *Leishmania* Cells. (i) Coomassie stained SDS-polyacrylamide gel (12%). (ii) Western blot of ‘i’ using anti-GFP antibodies. Lane 1: GFP-Myo21UBAs, lane 2: GFP-Myo21TΔ UBAs; Mr: molecular weight markers. Asterisk indicates GFP-Myo21UBAs band of size ~37kDa. Arrowhead indicates GFP-Myo21TΔUBAs band of size ~54.8kDa. (D) Growth analysis of wild type, GFP-Myo21UBAs and GFP-Myo21TΔUBAs expressing cells. The results are expressed as the means ± S. D. of three independent experiments.
(TIF)

S3 Fig Epiflourescence micrographs showing intraflagellar distributions. (A) Myo21-GFP, (B) Myo21ΔUBAs-GFP, (C) Myo21ΔUBA1-GFP and (D) Myo21ΔUBA2-GFP in *Leishmania* promastigotes. Scale bar—100 μm.
(TIF)

S4 Fig Co-localization of GFP fused proteins with actin. Immunofluorescence images of cells expressing (A) Myo21-GFP, (B) Myo21ΔUBAs-GFP, (C) Myo21ΔUBA1-GFP, and (D) Myo21ΔUBA2-GFP, labeled for actin (red). Myo21-GFP protein co-localizes with actin in the cell body, flagellum and also in the proximal region of the flagellum. However, Myo21ΔUBAs-GFP co-localized with actin in the cell body but virtually no co-distribution of these proteins could be seen in the flagellum, including its proximal region. Like Myo21ΔUBAs-GFP protein, Myo21ΔUBA1-GFP and Myo21ΔUBA2-GFP also failed to co-distribute with actin in the flagellum. Number of cells imaged for co-localization of GFP tagged protein with actin for Myo21-GFP- ~20, Myo21ΔUBAs-GFP—~18, Myo21ΔUBA1-GFP- ~19 and Myo21ΔUBA2-GFP- ~14 in at least three independent experiments. Arrowheads indicate co-distribution of Myo21-GFP with actin in the flagellum. Scale bar—2 μm.
(TIF)

S5 Fig Analysis of morphology of *Leishmania* cells expressing Myo21-GFP, Myo21ΔUBA1-GFP and Myo21ΔUBA2-GFP. (A) Analysis of the cell body length and width of wild type and Myo21-GFP expressing cells. (B) Histogram of flagellum lengths of wild type and Myo21-GFP expressing cells. (C) Analysis of the cell body length and width of Myo21-GFP, Myo21ΔUBA1-GFP and Myo21ΔUBA2-GFP expressing cells. (D) Histogram of flagellum lengths of Myo21-GFP, Myo21ΔUBA1-GFP and Myo21ΔUBA2-GFP expressing cells. ≥120 1N1K cells were measured for each cell type in three independent experiments.
(TIF)

S6 Fig Analysis of motility of *Leishmania* cells expressing Myo21ΔUBA1-GFP and Myo21ΔUBA2-GFP. Swimming tracks of (A) Myo21ΔUBA1-GFP and (B) Myo21ΔUBA2-GFP expressing cells from time-lapse video tracked using MTrack2 tracking tool in Fiji (ImageJ). Scale bar—100 μm. (C, D & E) Graphical representation of motility rate of Myo21ΔUBA1-GFP and Myo21ΔUBA2-GFP expressing cells relative to control cells. ≥30 cells were measured from at least three independent experiments for each cell type. The data were statistically analyzed by ANOVA test and a p-value of >0.05 was considered non-significant.
(TIF)

S7 Fig Analysis of intracellular trafficking activity of *Leishmania* cells expressing Myo21ΔUBA1-GFP and Myo21ΔUBA2-GFP. Endocytic internalization of FM4-64 in (A) Myo21ΔUBA1-GFP expressing cells and (B) Myo21ΔUBA2-GFP expressing cells. Cells were incubated with FM4-64FX for 10 min before washing and suspending in fresh medium. Thereafter, aliquots of cells were taken at 0 min, 30 min, 60 min and 120 min time point. Adhered and fixed cells were stained with DAPI (blue) to visualize nucleus (N) and kinetoplast (K); FM4-64 dye is in red. Scale bar—2 μm. (C). Quantitative analyses of Myo21ΔUBA1-GFP and Myo21ΔUBA2-GFP expressing cells showing percent of total cells which trafficked FM4-64 dye beyond the nucleus in 60 min (n = 43 and 36 for Myo21ΔUBA1-GFP and Myo21ΔUBA2-GFP expressing cells, respectively, from three independent experiments), compared to Myo21-GFP expressing cells.
(TIF)

S8 Fig Comparative flow cytometry analysis of hydroxy urea-synchronized Myo21-GFP and Myo21ΔUBAs-GFP expressing cells. After release of hydroxyurea pressure, at which time sampling was done is indicated on the right- hand side of the panel of histogram columns. 20,000 events were analyzed at every time-point. Three independent experiments were performed and one data-set is shown here. Arrows indicate G1, S and G2/M phases in histogram and arrowhead indicates sub-G1 phase (probably dead cell population).
(TIF)

S9 Fig Representative flow cytometry data of hydroxyurea-synchronized wild type cells, Myo21ΔUBA1-GFP and Myo21ΔUBA2-GFP expressing cells. 20,000 events were analyzed at every time-point. Myo21ΔUBA1-GFP and Myo21ΔUBA2-GFP expressing cells, similar to wild type cells, at 4 h have S phase maxima, at 6 h G2/M phase and at 8 h enter into the next G1 phase.
(TIF)

S10 Fig Graphical representation of cell cycle distribution. (A) Wild-type, (B) Myo21ΔUBA1-GFP and (C) Myo21ΔUBA2-GFP expressing cells, after removal of hydroxyurea (HU) block. Mid-log phase cells were synchronized by the HU treatment. DNA content was measured after staining with propidium iodide (PI) and flow cytometry analysis of cell cycle phases were done at every 2 h interval for up to 12 h. The percent of cells in each of the phase (G1 –circle, S–square and G2/M–triangles) at corresponding time point were calculated from the actual data using ModFit software. The results shown are means ± s. d. from three independent experiments.
(TIF)

S11 Fig Confocal microscopy images of *Leishmania* promastigotes expressing. (A) endogenous Myo21 only (control), (B) Myo21-GFP, (C) Myo21ΔUBAs-GFP, (D) Myo21ΔUBA1-GFP, (E) Myo21ΔUBA2-GFP, (F) GFP-Myo21UBAs, and (G) GFP-Myo21TΔUBAs, labeled for anti-Myo21 (green) and anti- α-tubulin (red) antibodies, and mounted in DAPI (blue) to visualize the DNA (nucleus and kinetoplast). Myosin localization at the base of the flagellum is visible in each of the construct expressing cells, as marked by the arrow. Scale bar—2 μm.
(TIF)

S12 Fig Immunofluorescence images of cells expressing. (A) Myo21-GFP, (B) Myo21ΔUBAs-GFP, (C) Myo21ΔUBA1-GFP, and (D) Myo21ΔUBA2-GFP, labeled for Myo21 (green) and actin (red), using ant-Myo21 and anti-LdAct antibodies. Myo21 protein co-localized with actin at the base of the flagellum in each of the construct expressing cells. Arrowheads indicate co-distribution of Myo21-GFP with actin in the flagellum. Scale bar—2 μm.
(TIF)

S1 Table List of primers used in the study. (DOCX)

S1 Movie (MP4)

S2 Movie (MP4)

S3 Movie (MP4)

S4 Movie (MP4)
