# Supplementary figures and images for: Deciphering the role of UBA-like domains in intraflagellar distribution and functions of myosin XXI in Leishmania
Source: PLoS One. 2020 Apr 28;15(4):e0232116. doi: 10.1371/journal.pone.0232116 (PMC7188243; doi:10.1371/journal.pone.0232116)

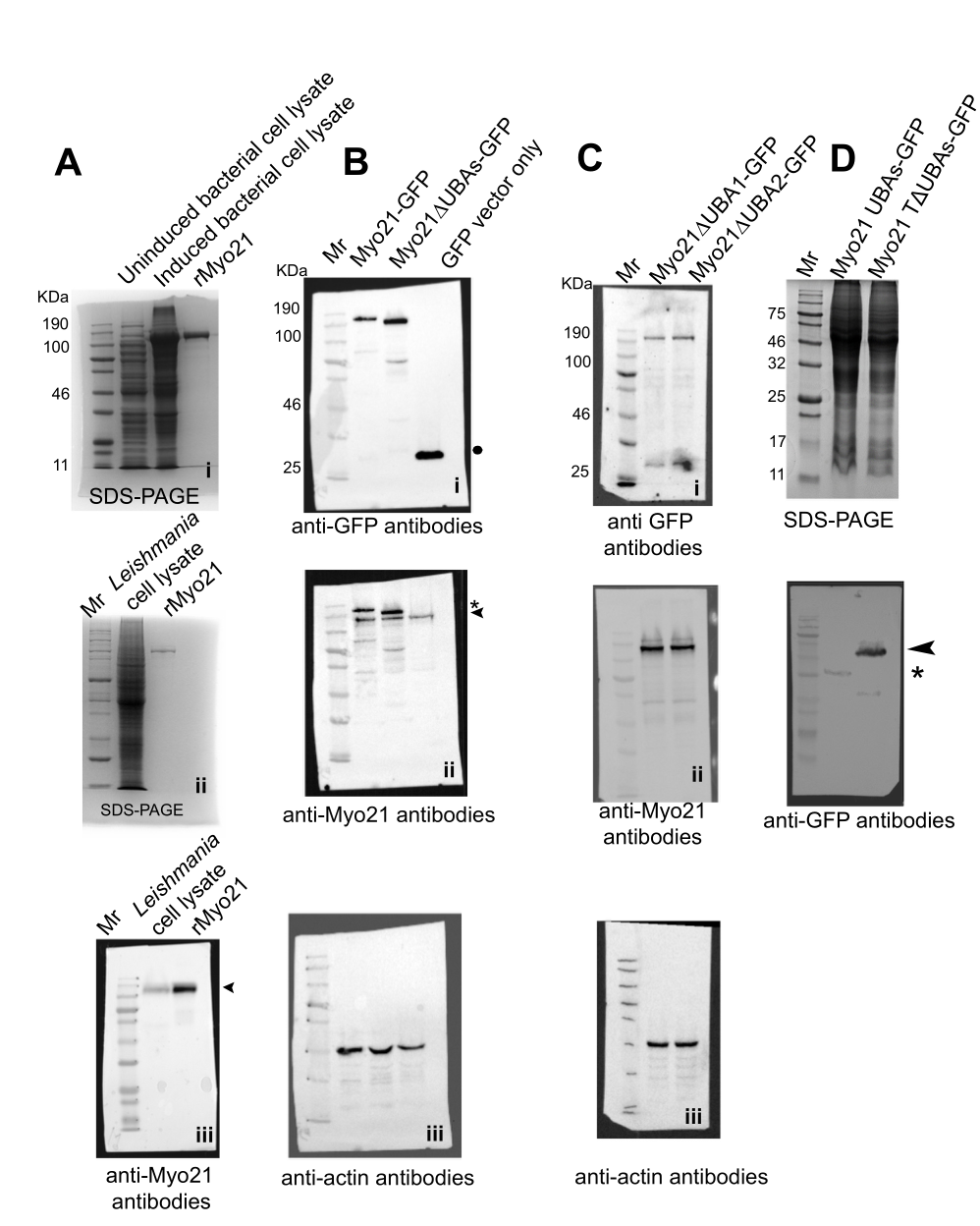

Supplement: S1 Fig — (TIFF) [file pone.0232116.s001.tiff]

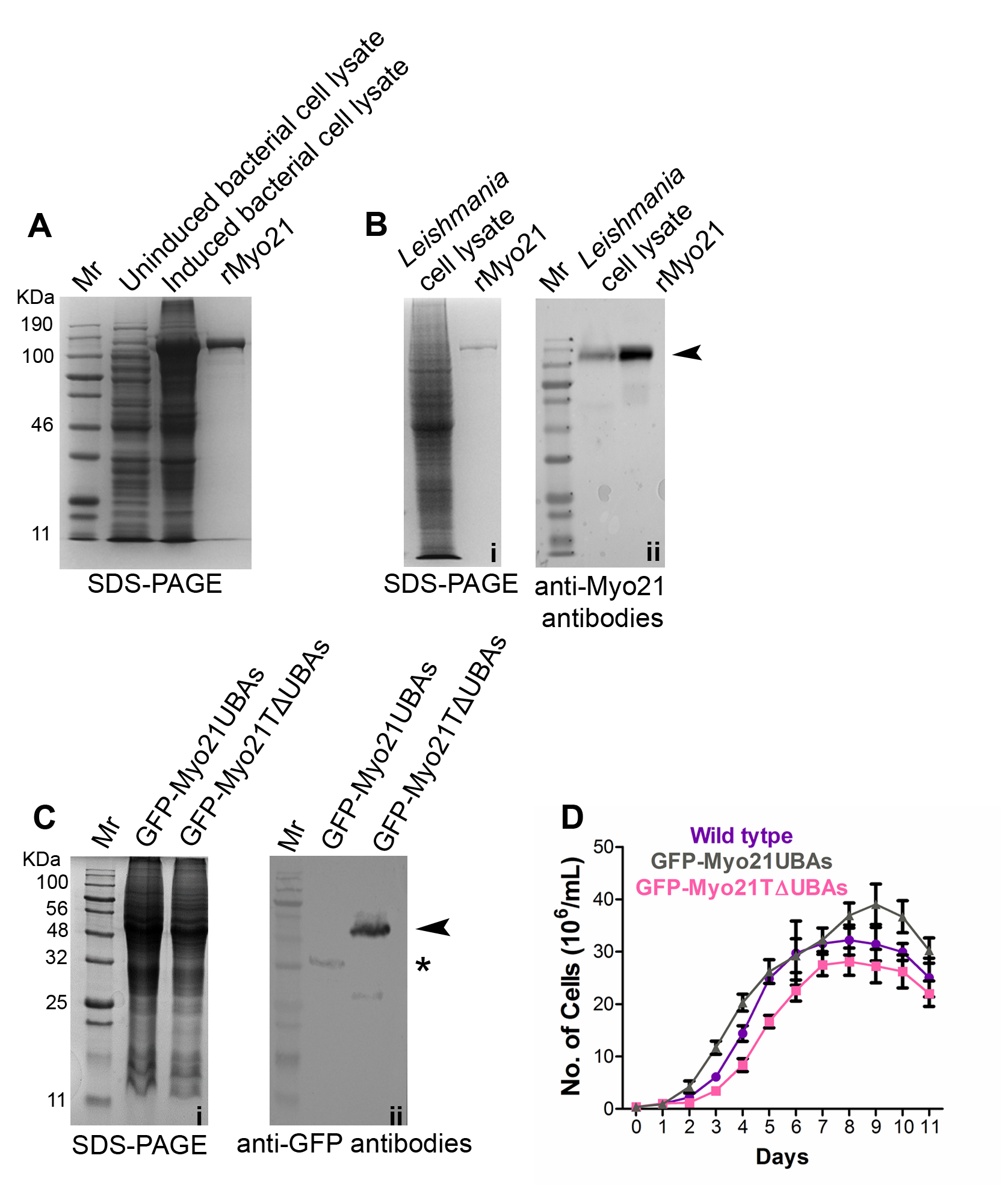

Supplement: S2 Fig — (A) Commassie stained SDS-polyacrylamide gel (10%) showing over-expressed Myo21 protein in bacterial cell lysate after IPTG induction and purified recombinant Myo21 (rMyo21) protein (~115KDa, arrowhead). Lane 1: Uninduced bacterial cell lysate, Lane 2: Induced bacterial cell lysate, Lane 3: rMyo21; Mr: molecular weight markers. Purified Myo21 protein was used for generation of antibodies. (B). Monospecific polyclonal Myo21 antibodies purified from rabbit serum were validated by western blotting, which detects a specific band of expected molecular weight ~115 KDa in Leishmania cell lysate (arrowhead). (i) Commassie stained SDS-polyacrylamide gel (10%). (ii) Western blot of using purified Myo21 antibodies. Lane 1: Leishmania cell lysate, lane 2: rMyo21; Mr: molecular weight markers. (C) Expression of Myo21UBAs-GFPand Myo21TΔUBAs-GFP in Leishmania Cells. (i) Coomassie stained SDS-polyacrylamide gel (12%). (ii) Western blot of ‘i’ using anti-GFP antibodies. Lane 1: GFP-Myo21UBAs, lane 2: GFP-Myo21TΔ UBAs; Mr: molecular weight markers. Asterisk indicates GFP-Myo21UBAs band of size ~37kDa. Arrowhead indicates GFP-Myo21TΔUBAs band of size ~54.8kDa. (D) Growth analysis of wild type, GFP-Myo21UBAs and GFP-Myo21TΔUBAs expressing cells. The results are expressed as the means ± S. D. of three independent experiments. (TIF) [file pone.0232116.s002.tif]

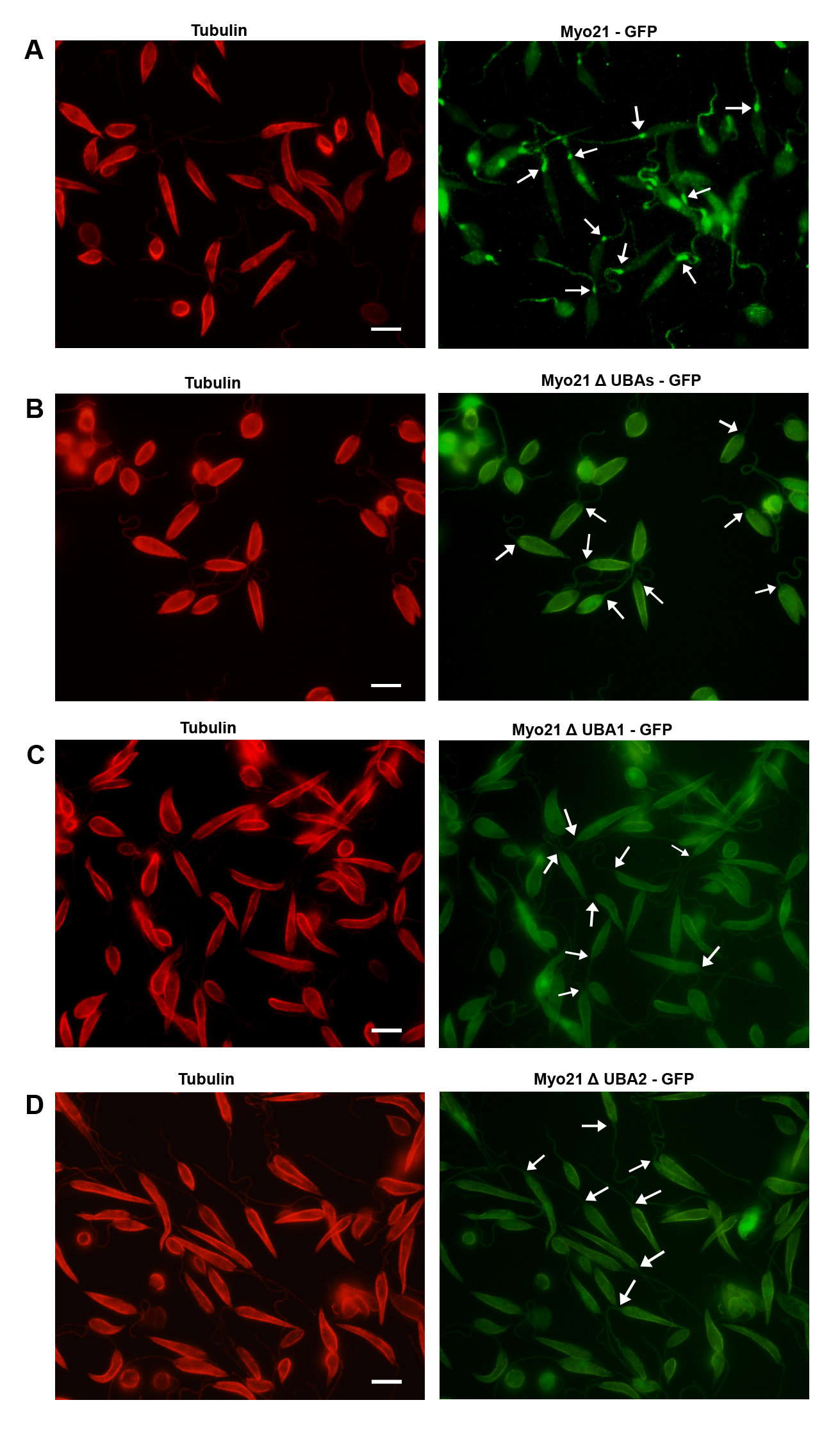

Supplement: S3 Fig — (A) Myo21-GFP, (B) Myo21ΔUBAs-GFP, (C) Myo21ΔUBA1-GFP and (D) Myo21ΔUBA2-GFP in Leishmania promastigotes. Scale bar—100 μm. (TIF) [file pone.0232116.s003.tif]

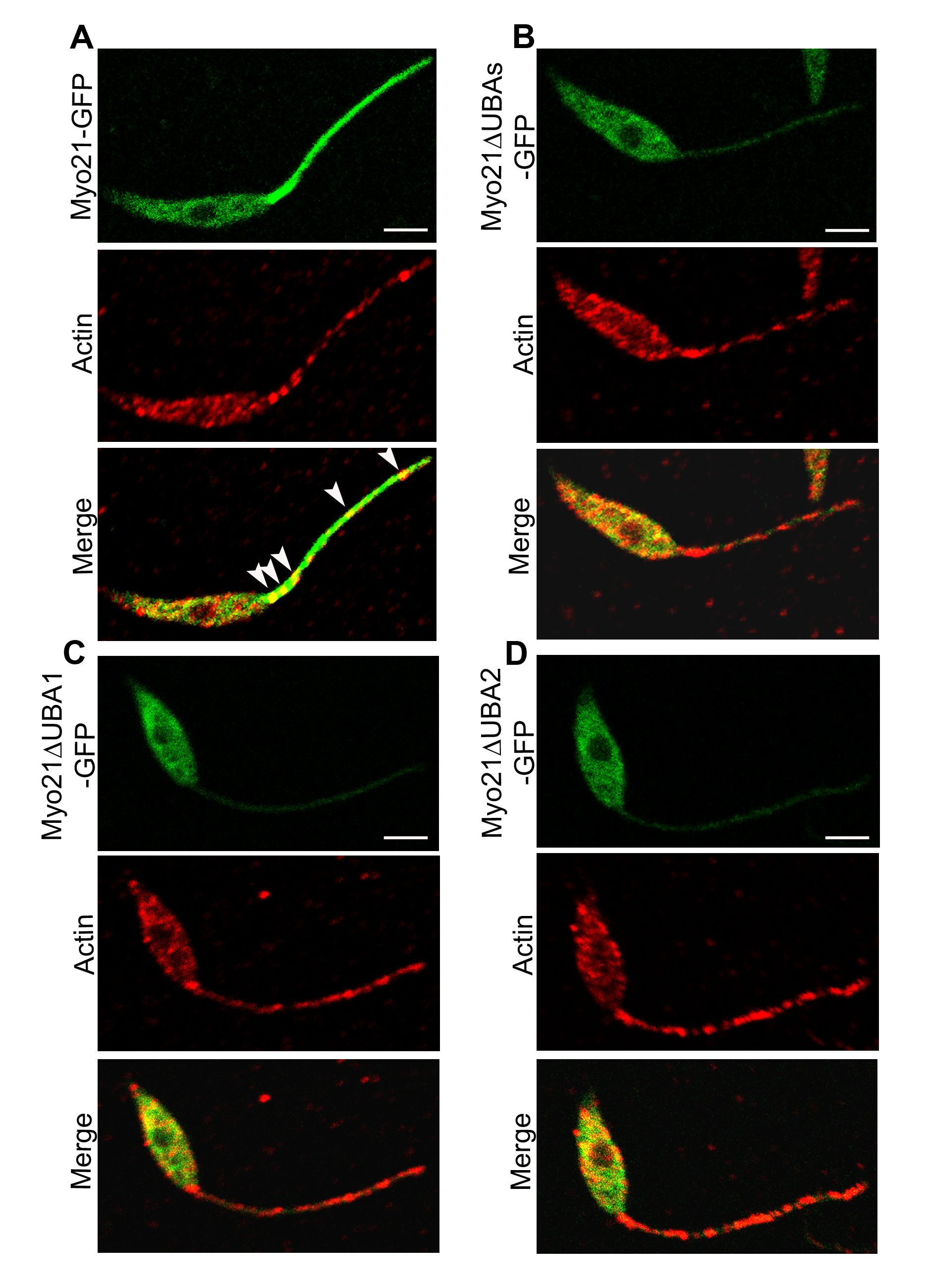

Supplement: S4 Fig — Immunofluorescence images of cells expressing (A) Myo21-GFP, (B) Myo21ΔUBAs-GFP, (C) Myo21ΔUBA1-GFP, and (D) Myo21ΔUBA2-GFP, labeled for actin (red). Myo21-GFP protein co-localizes with actin in the cell body, flagellum and also in the proximal region of the flagellum. However, Myo21ΔUBAs-GFP co-localized with actin in the cell body but virtually no co-distribution of these proteins could be seen in the flagellum, including its proximal region. Like Myo21ΔUBAs-GFP protein, Myo21ΔUBA1-GFP and Myo21ΔUBA2-GFP also failed to co-distribute with actin in the flagellum. Number of cells imaged for co-localization of GFP tagged protein with actin for Myo21-GFP- ~20, Myo21ΔUBAs-GFP—~18, Myo21ΔUBA1-GFP- ~19 and Myo21ΔUBA2-GFP- ~14 in at least three independent experiments. Arrowheads indicate co-distribution of Myo21-GFP with actin in the flagellum. Scale bar—2 μm. (TIF) [file pone.0232116.s004.tif]

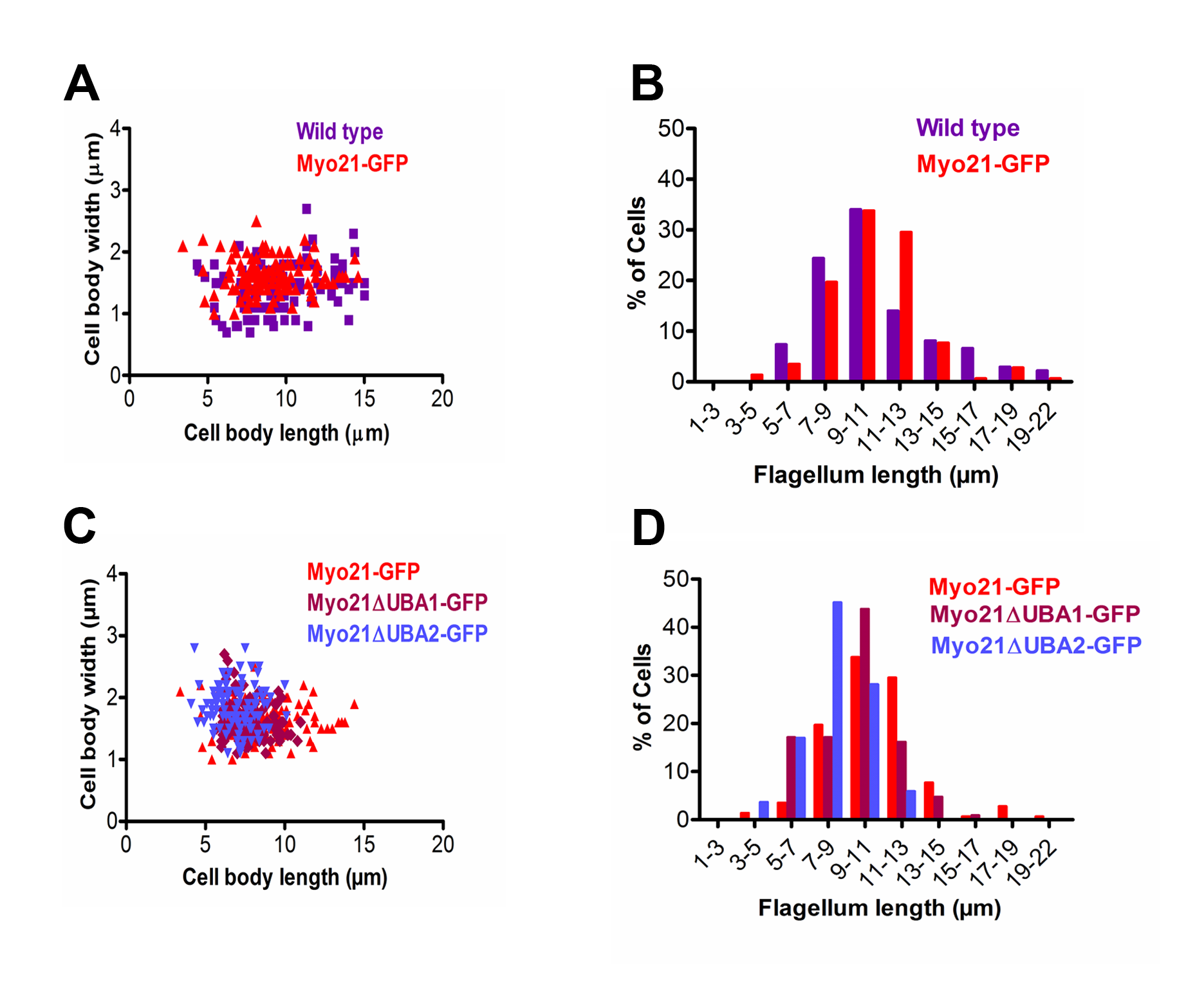

Supplement: S5 Fig — (A) Analysis of the cell body length and width of wild type and Myo21-GFP expressing cells. (B) Histogram of flagellum lengths of wild type and Myo21-GFP expressing cells. (C) Analysis of the cell body length and width of Myo21-GFP, Myo21ΔUBA1-GFP and Myo21ΔUBA2-GFP expressing cells. (D) Histogram of flagellum lengths of Myo21-GFP, Myo21ΔUBA1-GFP and Myo21ΔUBA2-GFP expressing cells. ≥120 1N1K cells were measured for each cell type in three independent experiments. (TIF) [file pone.0232116.s005.tif]

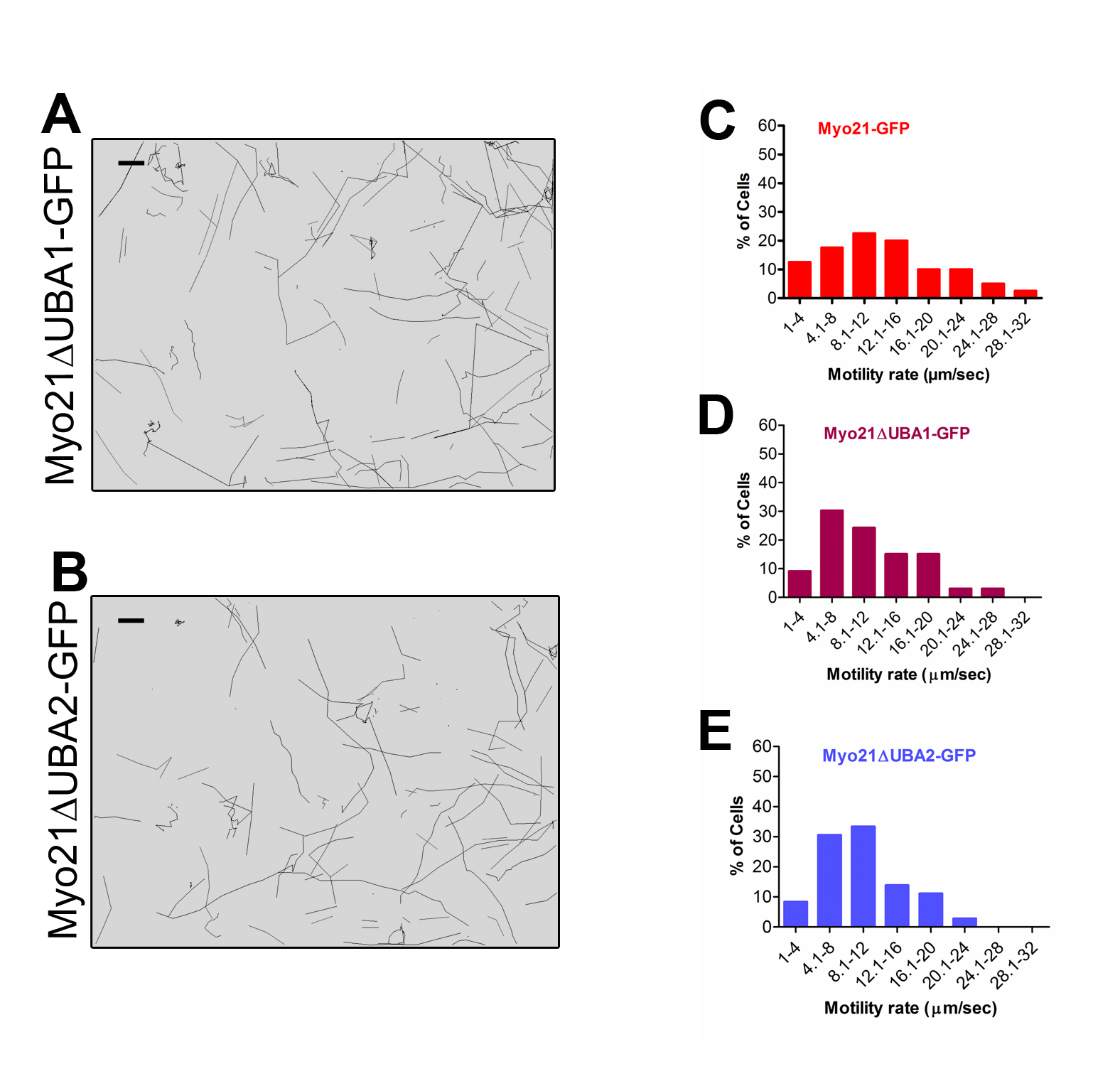

Supplement: S6 Fig — Swimming tracks of (A) Myo21ΔUBA1-GFP and (B) Myo21ΔUBA2-GFP expressing cells from time-lapse video tracked using MTrack2 tracking tool in Fiji (ImageJ). Scale bar—100 μm. (C, D & E) Graphical representation of motility rate of Myo21ΔUBA1-GFP and Myo21ΔUBA2-GFP expressing cells relative to control cells. ≥30 cells were measured from at least three independent experiments for each cell type. The data were statistically analyzed by ANOVA test and a p-value of >0.05 was considered non-significant. (TIF) [file pone.0232116.s006.tif]

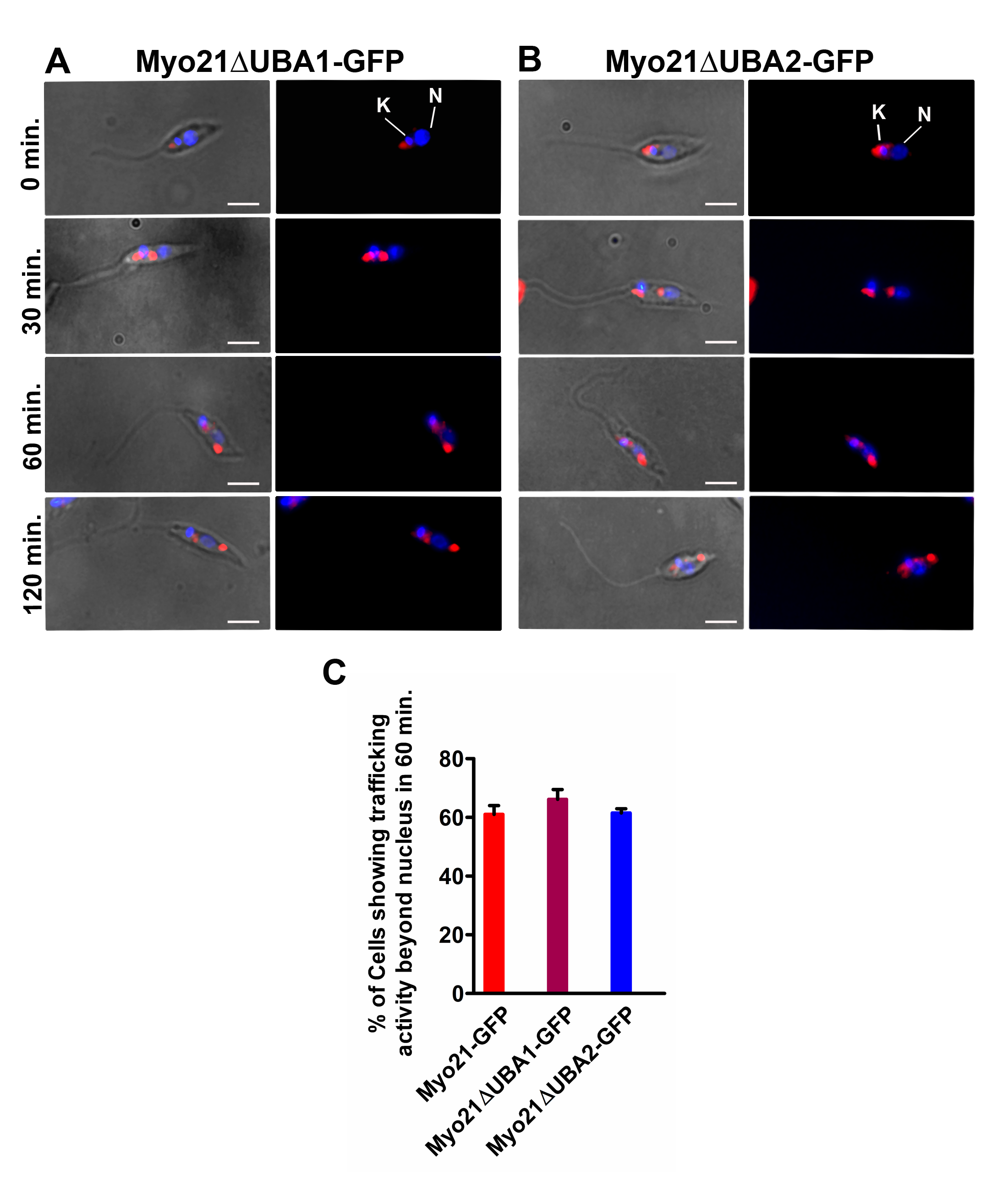

Supplement: S7 Fig — Endocytic internalization of FM4-64 in (A) Myo21ΔUBA1-GFP expressing cells and (B) Myo21ΔUBA2-GFP expressing cells. Cells were incubated with FM4-64FX for 10 min before washing and suspending in fresh medium. Thereafter, aliquots of cells were taken at 0 min, 30 min, 60 min and 120 min time point. Adhered and fixed cells were stained with DAPI (blue) to visualize nucleus (N) and kinetoplast (K); FM4-64 dye is in red. Scale bar—2 μm. (C). Quantitative analyses of Myo21ΔUBA1-GFP and Myo21ΔUBA2-GFP expressing cells showing percent of total cells which trafficked FM4-64 dye beyond the nucleus in 60 min (n = 43 and 36 for Myo21ΔUBA1-GFP and Myo21ΔUBA2-GFP expressing cells, respectively, from three independent experiments), compared to Myo21-GFP expressing cells. (TIF) [file pone.0232116.s007.tif]

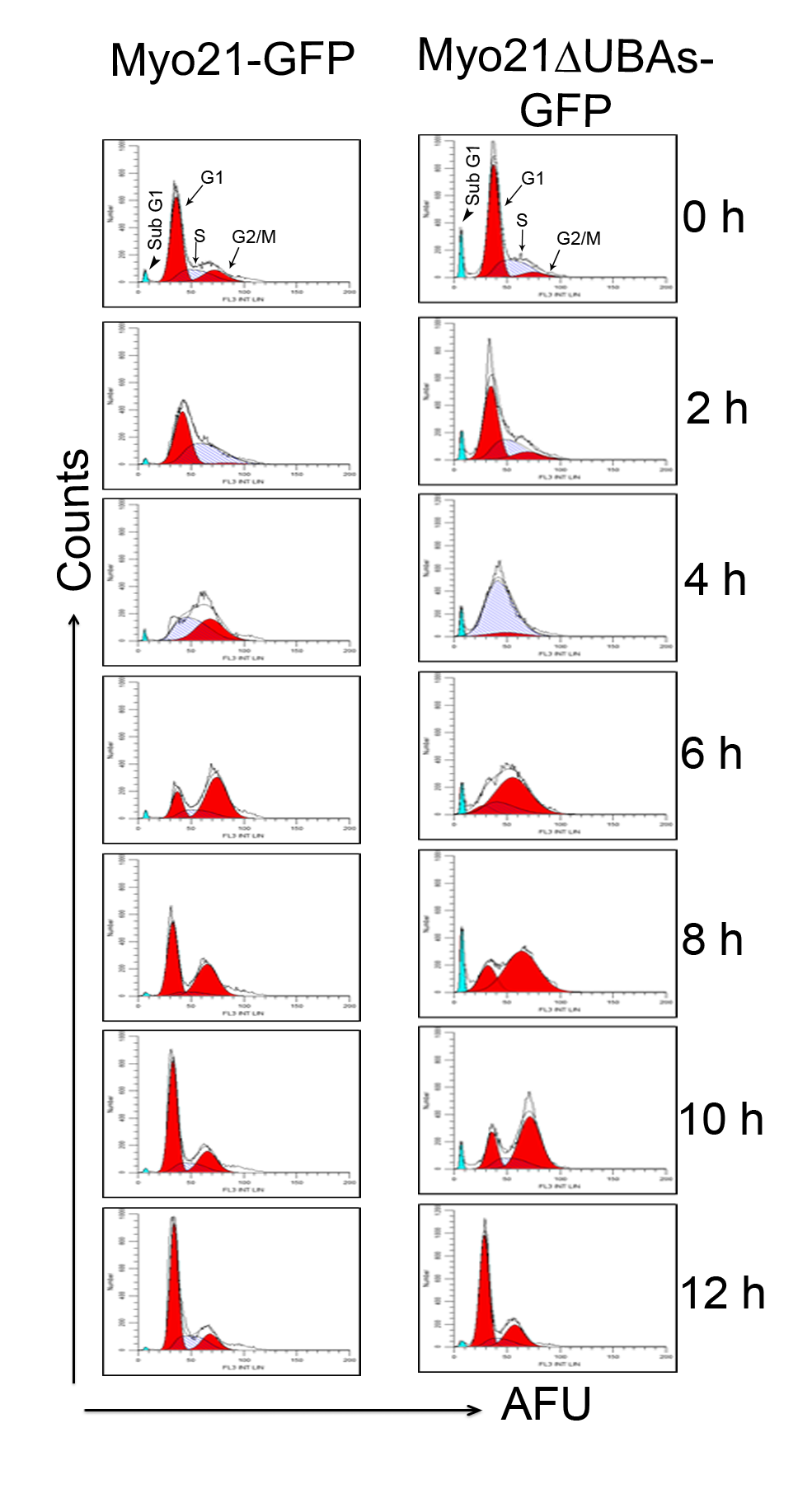

Supplement: S8 Fig — After release of hydroxyurea pressure, at which time sampling was done is indicated on the right- hand side of the panel of histogram columns. 20,000 events were analyzed at every time-point. Three independent experiments were performed and one data-set is shown here. Arrows indicate G1, S and G2/M phases in histogram and arrowhead indicates sub-G1 phase (probably dead cell population). (TIF) [file pone.0232116.s008.tif]

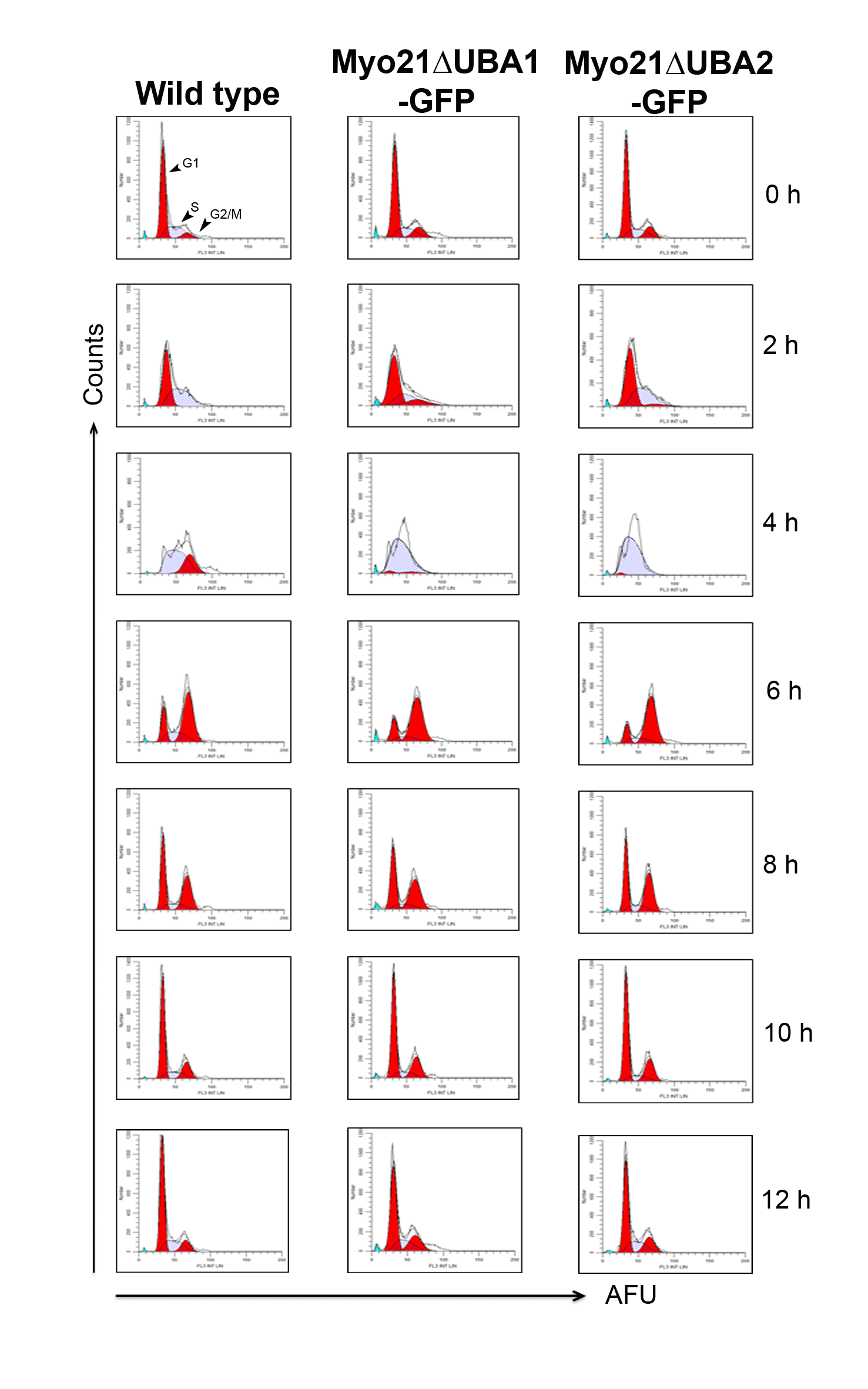

Supplement: S9 Fig — 20,000 events were analyzed at every time-point. Myo21ΔUBA1-GFP and Myo21ΔUBA2-GFP expressing cells, similar to wild type cells, at 4 h have S phase maxima, at 6 h G2/M phase and at 8 h enter into the next G1 phase. (TIF) [file pone.0232116.s009.tif]

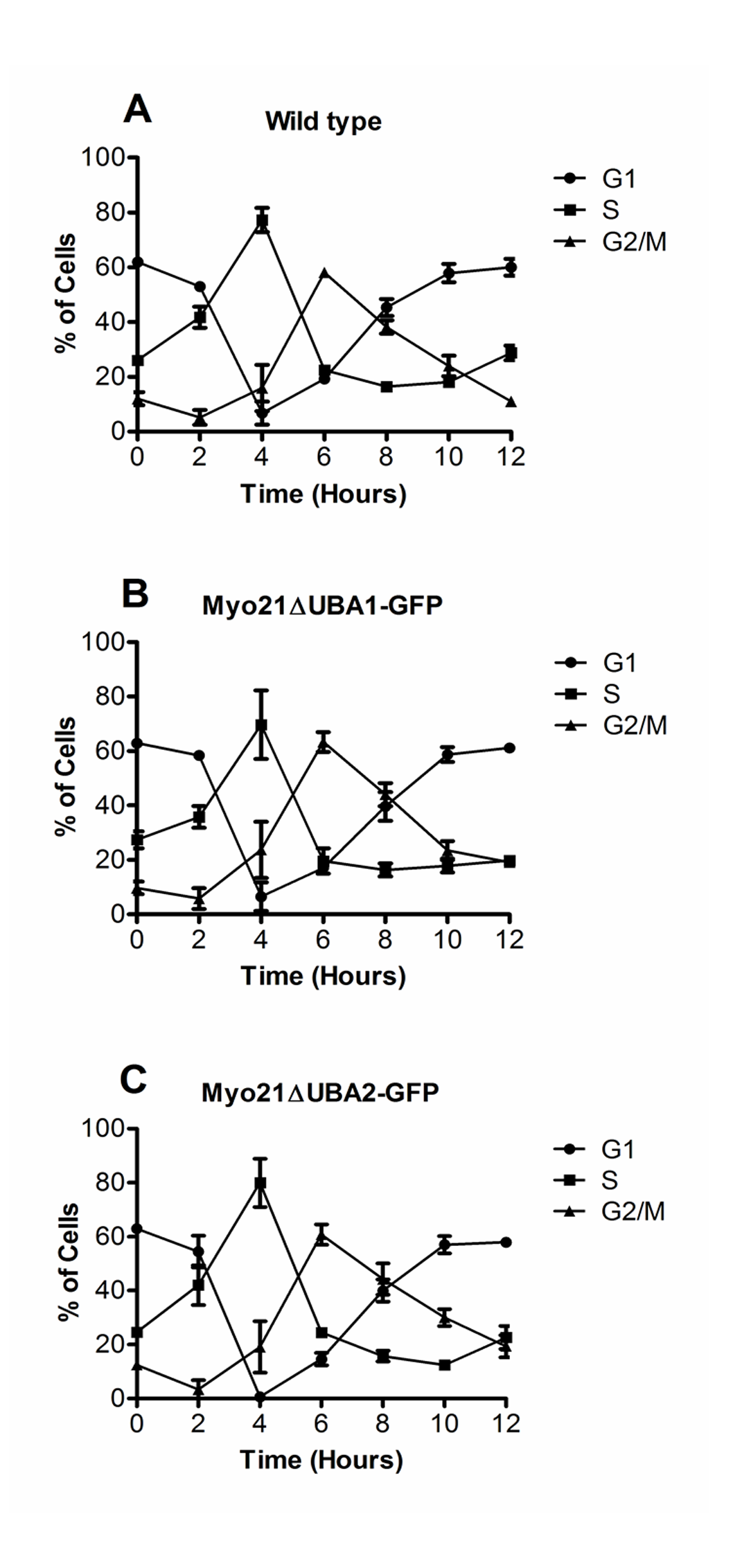

Supplement: S10 Fig — (A) Wild-type, (B) Myo21ΔUBA1-GFP and (C) Myo21ΔUBA2-GFP expressing cells, after removal of hydroxyurea (HU) block. Mid-log phase cells were synchronized by the HU treatment. DNA content was measured after staining with propidium iodide (PI) and flow cytometry analysis of cell cycle phases were done at every 2 h interval for up to 12 h. The percent of cells in each of the phase (G1 –circle, S–square and G2/M–triangles) at corresponding time point were calculated from the actual data using ModFit software. The results shown are means ± s. d. from three independent experiments. (TIF) [file pone.0232116.s010.tif]

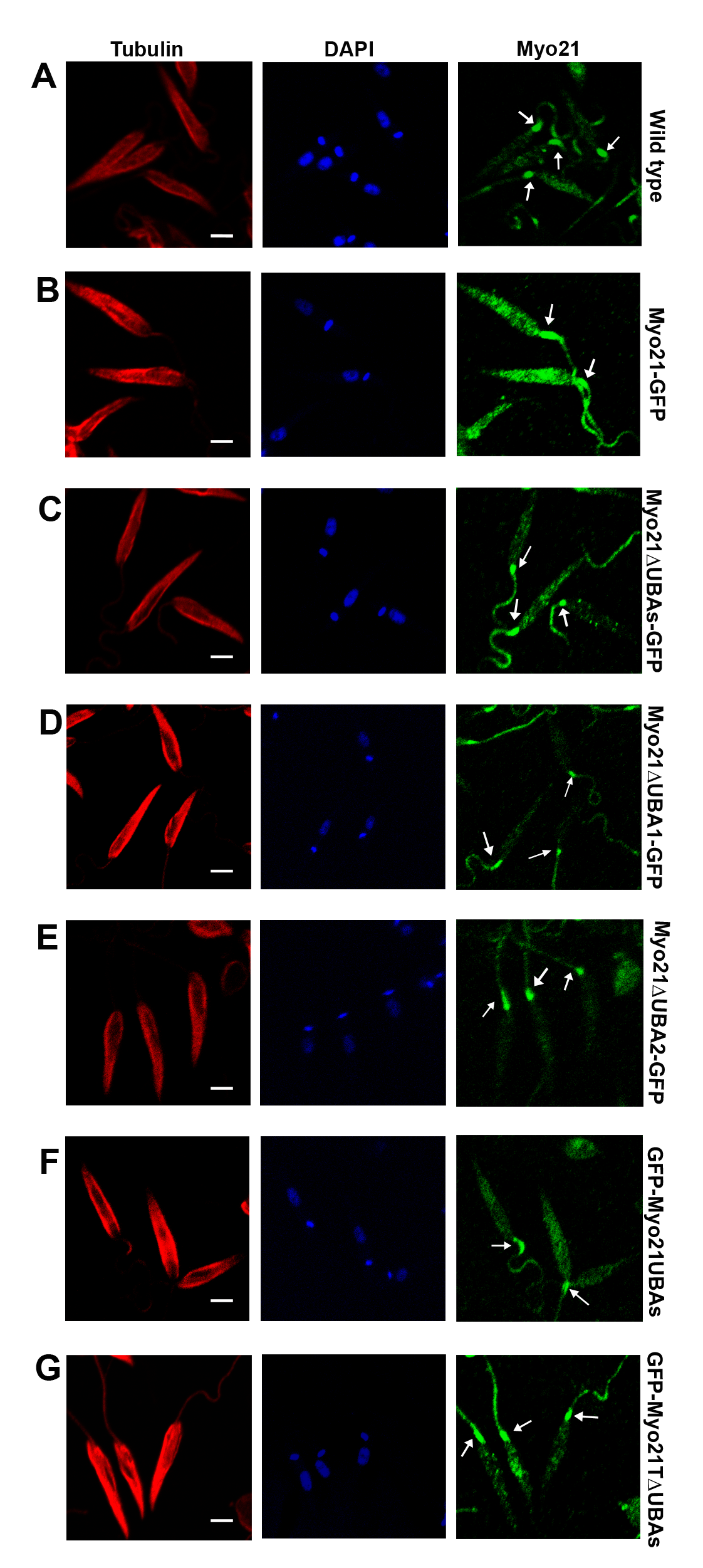

Supplement: S11 Fig — (A) endogenous Myo21 only (control), (B) Myo21-GFP, (C) Myo21ΔUBAs-GFP, (D) Myo21ΔUBA1-GFP, (E) Myo21ΔUBA2-GFP, (F) GFP-Myo21UBAs, and (G) GFP-Myo21TΔUBAs, labeled for anti-Myo21 (green) and anti- α-tubulin (red) antibodies, and mounted in DAPI (blue) to visualize the DNA (nucleus and kinetoplast). Myosin localization at the base of the flagellum is visible in each of the construct expressing cells, as marked by the arrow. Scale bar—2 μm. (TIF) [file pone.0232116.s011.tif]

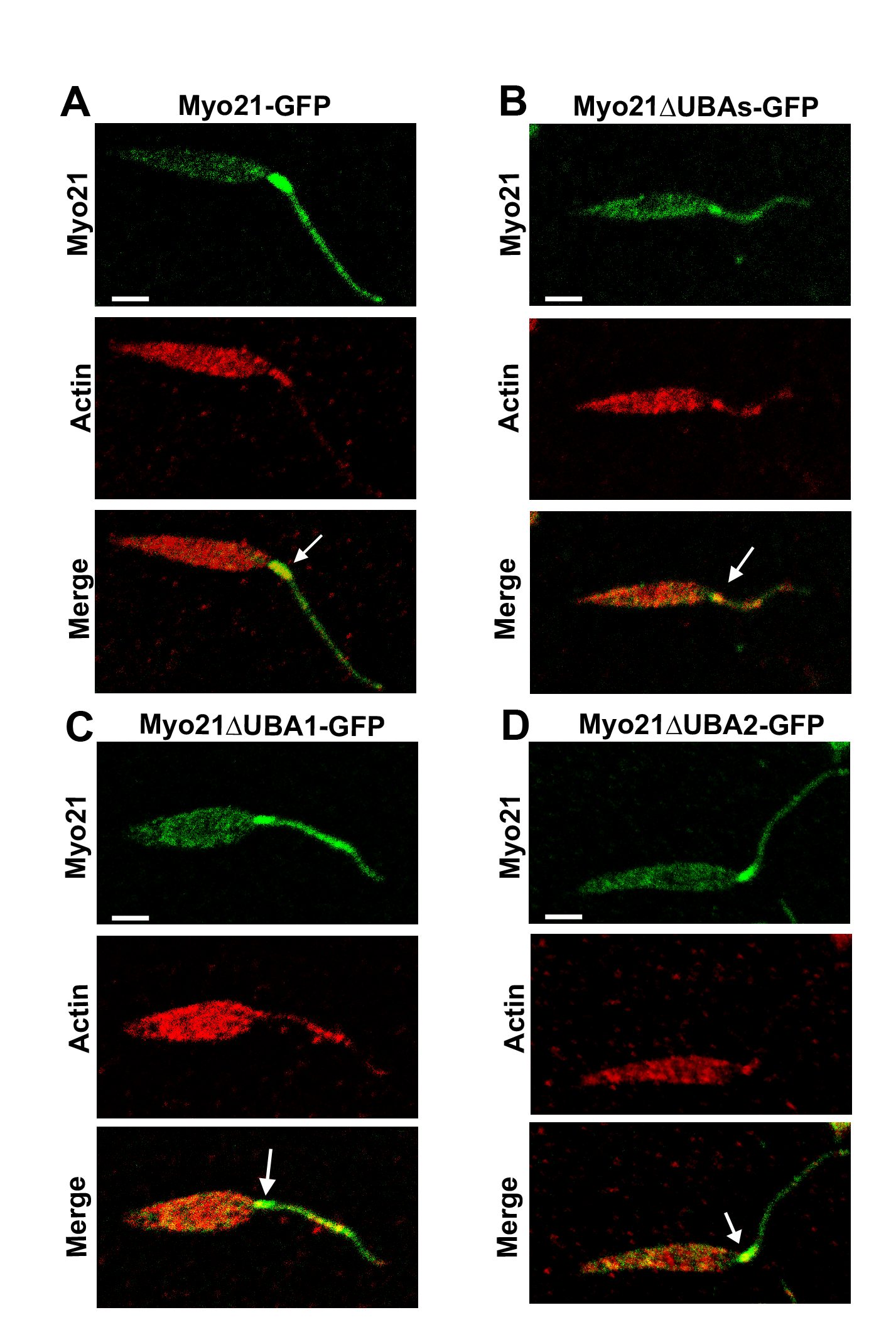

Supplement: S12 Fig — (A) Myo21-GFP, (B) Myo21ΔUBAs-GFP, (C) Myo21ΔUBA1-GFP, and (D) Myo21ΔUBA2-GFP, labeled for Myo21 (green) and actin (red), using ant-Myo21 and anti-LdAct antibodies. Myo21 protein co-localized with actin at the base of the flagellum in each of the construct expressing cells. Arrowheads indicate co-distribution of Myo21-GFP with actin in the flagellum. Scale bar—2 μm. (TIF) [file pone.0232116.s012.tif]
