# Supplementary material for: Deciphering the role of UBA-like domains in intraflagellar distribution and functions of myosin XXI in Leishmania
Source: PLoS One. 2020 Apr 28;15(4):e0232116. doi: 10.1371/journal.pone.0232116 (PMC7188243; doi:10.1371/journal.pone.0232116)
Supplement: S1 Table — (DOCX) [file pone.0232116.s013.docx]

**Table S1 – List of primers used in the study.**

| F1 | 5’GTATAGGATCCATGCCGGAGCGCGTGTCTGTG 3’ |
| --- | --- |
| ­­­­R1 | 5’GCACTGGATCCGCTCACCTTGAACAGCATCTTAGCG 3’­­­ |
| R2 | 5’ GGATCGGATCCGTCTTCCTGGTGGCGAGC 3’ |
| F2 | 5’ GCTCGCCACCAGGAAGAC CGCCTGCTTTATGGCGTG 3’ |
| R3 | 5’ CACGCCATAAAGCAGGCG GTCTTCCTGGTGGCGAGC 3’ |
| R4 | 5’ GTCGGGGATCCCGTTGTGTTGAAGTTCTTG 3’ |
| F3 | 5’ GGCCCGGATCCTTCGACGCCAAAGTGCAGACC 3’ |
| R5 | 5’ GGCCCGGATCCCTAGCTCACCTTGAACAGCATCTTAGCG 3’ |
| F4 | 5’ TAATAGGATCCCGTCGCCGCGCGCAGCTG 3’ |
| R6 | 5’ GGCCCGGATCCCTAGTCTTCCTGGTGGCGAGC 3’ |
